# Children’s role in the COVID-19 pandemic: a systematic review of early surveillance data on susceptibility, severity, and transmissibility

**DOI:** 10.1038/s41598-021-92500-9

**Published:** 2021-07-06

**Authors:** Katy A. M. Gaythorpe, Sangeeta Bhatia, Tara Mangal, H. Juliette T. Unwin, Natsuko Imai, Gina Cuomo-Dannenburg, Caroline E. Walters, Elita Jauneikaite, Helena Bayley, Mara D. Kont, Andria Mousa, Lilith K. Whittles, Steven Riley, Neil M. Ferguson

**Affiliations:** 1 MRC Centre for Global Infectious Disease Analysis and WHO Collaborating Centre for Infectious Disease Modelling, Abdul Latif Jameel Institute for Disease and Emergency Analytics, Imperial College London, London, UK; 2 Department of Physics, University of Oxford, Oxford, UK

**Keywords:** Infectious diseases, Viral infection

## Abstract

SARS-CoV-2 infections have been reported in all age groups including infants, children, and adolescents. However, the role of children in the COVID-19 pandemic is still uncertain. This systematic review of early studies synthesises evidence on the susceptibility of children to SARS-CoV-2 infection, the severity and clinical outcomes in children with SARS-CoV-2 infection, and the transmissibility of SARS-CoV-2 by children in the initial phases of the COVID-19 pandemic. A systematic literature review was conducted in PubMed. Reviewers extracted data from relevant, peer-reviewed studies published up to July 4th 2020 during the first wave of the SARS-CoV-2 outbreak using a standardised form and assessed quality using the NIH Quality Assessment Tool for Observational Cohort and Cross-Sectional Studies. For studies included in the meta-analysis, we used a random effects model to calculate pooled estimates of the proportion of children considered asymptomatic or in a severe or critical state. We identified 2775 potential studies of which 128 studies met our inclusion criteria; data were extracted from 99, which were then quality assessed. Finally, 29 studies were considered for the meta-analysis that included information of symptoms and/or severity, these were further assessed based on patient recruitment. Our pooled estimate of the proportion of test positive children who were asymptomatic was 21.1% (95% CI: 14.0–28.1%), based on 13 included studies, and the proportion of children with severe or critical symptoms was 3.8% (95% CI: 1.5–6.0%), based on 14 included studies. We did not identify any studies designed to assess transmissibility in children and found that susceptibility to infection in children was highly variable across studies. Children’s susceptibility to infection and onward transmissibility relative to adults is still unclear and varied widely between studies. However, it is evident that most children experience clinically mild disease or remain asymptomatically infected. More comprehensive contact-tracing studies combined with serosurveys are needed to quantify children’s transmissibility relative to adults. With children back in schools, testing regimes and study protocols that will allow us to better understand the role of children in this pandemic are critical.

## Introduction

Cases of atypical pneumonia were first reported in Wuhan City, China in late 2019. Since then, SARS-CoV-2 has spread rapidly across the globe and the World Health Organization declared COVID-19 a pandemic on March 11, 2020^1^. As of May 12, 2021 there have been a total 158,685,909 reported cases and 3,299,666 deaths^2^. In the absence of effective therapeutics or vaccines early in the pandemic, countries implemented a range of non-pharmaceutical interventions (NPIs) to limit the spread of the virus.

Based on what was known about the role of children in the spread of influenza during previous pandemics, school closures were widely implemented with an estimated 80–100% of EU/EEA countries closing pre-schools, primary and secondary schools, and higher education institutions as the pandemic progressed from February 2020 in East Asia to late April across Europe and the Americas^3–5^. Widespread NPIs and effective “lockdowns”, many including school-closures, have had a substantial role in suppressing transmission^6^. However, changes in contact patterns due to these measures have made it difficult to understand the extent to which children were infected by SARS-CoV-2 or were able to transmit SARS-CoV-2 onto others. Children and young adults represent a small proportion of the total reported cases of COVID-19 globally thus far, but the fraction of severe cases and deaths amongst this age group was small^7^. There was evidence^7–12^ for the age-dependency in clinical cases with severe cases and deaths concentrated in the elderly and amongst those with comorbidities in the early phase of COVID-19 pandemic. However, while there is consensus that children generally have milder clinical symptoms^7,13,14^, it is important to note that early routine testing and diagnosis were restricted mainly to symptomatic cases requiring health care in most countries. Therefore, data reported by countries through routine surveillance systems early in the pandemic were likely to significantly under-estimate cases amongst those with mild or no symptoms.

In addition, the widespread social distancing (SD) measures, such as restrictions on gatherings, encouragement to work from home, and closures of bars and restaurants, have substantially impacted contact patterns between individuals^15^ and universal school-closures would have had significantly affected how much children interact with others. At the beginning of the pandemic, outbreaks of COVID-19 in schools had not been widely reported. However, national holidays and school closures in response to the pandemic would have changed the risk of exposure to SARS-CoV-2 infection by reducing the number of non-household contacts that children have. It has been suggested that children are less susceptible to SARS-CoV-2 infection due to differences in their innate immune system, which means a faster and broader immune response can be mounted^16^. However, a flat attack rate has been observed across different age groups in contact-tracing studies^17^, population-based infection surveys^18^ and retrospective seroprevalence surveys^19,20^. This is typical of a novel pathogen in a population with no prior immunity. There is also increasing evidence that the age-distribution of newly detected cases is shifting towards younger age groups which may been captured as a result of the expanded test capacity in many countries including the UK and USA compared to April 2020^21^. This shift has now starting to be reflected in increased hospitalisations amongst young adults (20–35 years old), but whether children will also be affected at the same rate is yet to be seen^22^.

The biggest uncertainty is whether children are intrinsically less infectious than adults and how much they contribute to onward transmission. Within household clusters, children are not often identified as the index case, but this is difficult to determine due to the age-dependent clinical symptoms and a larger proportion of infections in children appearing to be asymptomatic^23^. The correlation between clinical severity and transmissibility is also difficult to disentangle, since pre-symptomatic transmission has been shown to be an important driver of transmission within the population^24–26^. It is unclear to what extent mild and asymptomatic cases also contribute to onward transmission at any age^27^.

Policies around schools are being adjusted based on new evidence and the level of transmission in each country. With growing concern about the long-term developmental and mental health impacts of school closures on children, the safe re-opening of schools has become a priority across different governments. Understanding the role of children in SARS-CoV-2 transmission is therefore critical in guiding ongoing policy for schools conducting face-to-face education.

In this study we undertook a systematic review of the peer-reviewed literature, excluding pre-prints, to synthesize available data on three specific topics: (i) the susceptibility of children to SARS-CoV-2 infection; (ii) the severity and clinical outcomes in children with SARS-CoV-2 infection; and (iii) the transmissibility of SARS-CoV-2 by children. We conducted a meta-analysis of data collected to address these topics and discuss their implications for future public health policies. We limited this review to the early stages of the epidemic, reflecting the first pieces of evidence generated during an emerging outbreak, where large population serosurveys and extensive contact tracing were uncommon.

## Methods

### Literature search

Our methods adhere to the guidelines established by Preferred Reporting Items for Systematic Reviews and Meta-Analyses (PRISMA). Our study protocol was registered with PROSPERO (International Prospective Register of Systematic Reviews) under the identifier #CRD42020184605.

We searched PubMed for "(*sars-cov-2* OR *nCov* OR *covid-19*) AND *children* AND (*infection* or *susceptibility* or *transmission* or *shedding* or *symptomatic*)". As SARS-CoV-2 is a new human pathogen, we restricted our searches to records from 2019 and 2020. The initial search was conducted on May 6, 2020 and repeated on July 4, 2020. All records were imported into Covidence (v2014, accessed 2020), where duplicates were removed. Both the titles/abstract and full-text screening were conducted by at least two independent reviewers and any conflicts resolved by consensus. Cohort studies, contact-tracing studies and population surveys were included. We excluded: (i) non-peer reviewed studies; (ii) case reports reporting fewer than 10 cases due to the high potential for bias and limited amount of information; (iii) papers focusing on vertical transmission; and (iv) non-English language papers. Full inclusion and exclusion criteria can be found in the Supplementary Information ("Inclusion and exclusion criteria" section).

Outcomes of interest were the risk in children of infection with COVID-19 following exposure i.e. aged distributed attack rate (up to 18 years of age); the risk of onward transmission of COVID-19 to contacts of an infected child i.e. secondary attack rates from a paediatric index case; and the proportion of symptomatic children and their disease severity (defined as mild disease/asymptomatic or severe disease requiring hospital care) of all children with confirmed COVID-19 infection.

### Data extraction and quality assessment

Data extraction was performed by all investigators using an Excel spreadsheet. Data collected included the type of study (e.g. cohort), country of origin, number of patients or size of cohort considered, demographic information (including age and sex where available), clinical symptoms and severity, and seroprevalence or attack rates where available (Supplementary Information Table S1).

The methodological quality of included studies was assessed by two reviewers using 10-item questions, which we adapted from the NIH Quality Assessment Tool for Observational Cohort and Cross-Sectional Studies^28^ to assess the strength of evidence relating to our research questions specifically e.g. assessing if the case definitions were clearly specified in the study (Supplementary Information Tables S2 and S3). Studies that scored 7 or more points were classified as good, those that scored 5 or 6 points were rated as fair, and any study that scored fewer than 5 points was rated as poor at informing our research questions. Only studies rated fair or good were included in the meta-analysis, these were also further assessed using the criterion detailed in the supplementary material.

### Data analysis

All extracted studies were filtered by topic, so data analysis was conducted only on studies including details on the quantity of interest, for example the number of individuals classed as asymptomatic. For the purposes of our analysis, we defined children as individuals aged 18 years or younger. The filtered studies were then checked for comparability, based on study type. Random effects modelling was performed using R package *metaphor*^29^ to produce estimates of the proportion of children considered asymptomatic or in a severe/critical state. We used an empirical Bayes estimator for the level of heterogeneity and weighted by the size of the study population. Analyses were conducted using R package *orderly* version 1.1.29^30^ in R version 3.6.3. Data and code are available from https://github.com/mrc-ide/child_covid19_lit_review.

## Results

### Literature search

**Figure 1:**
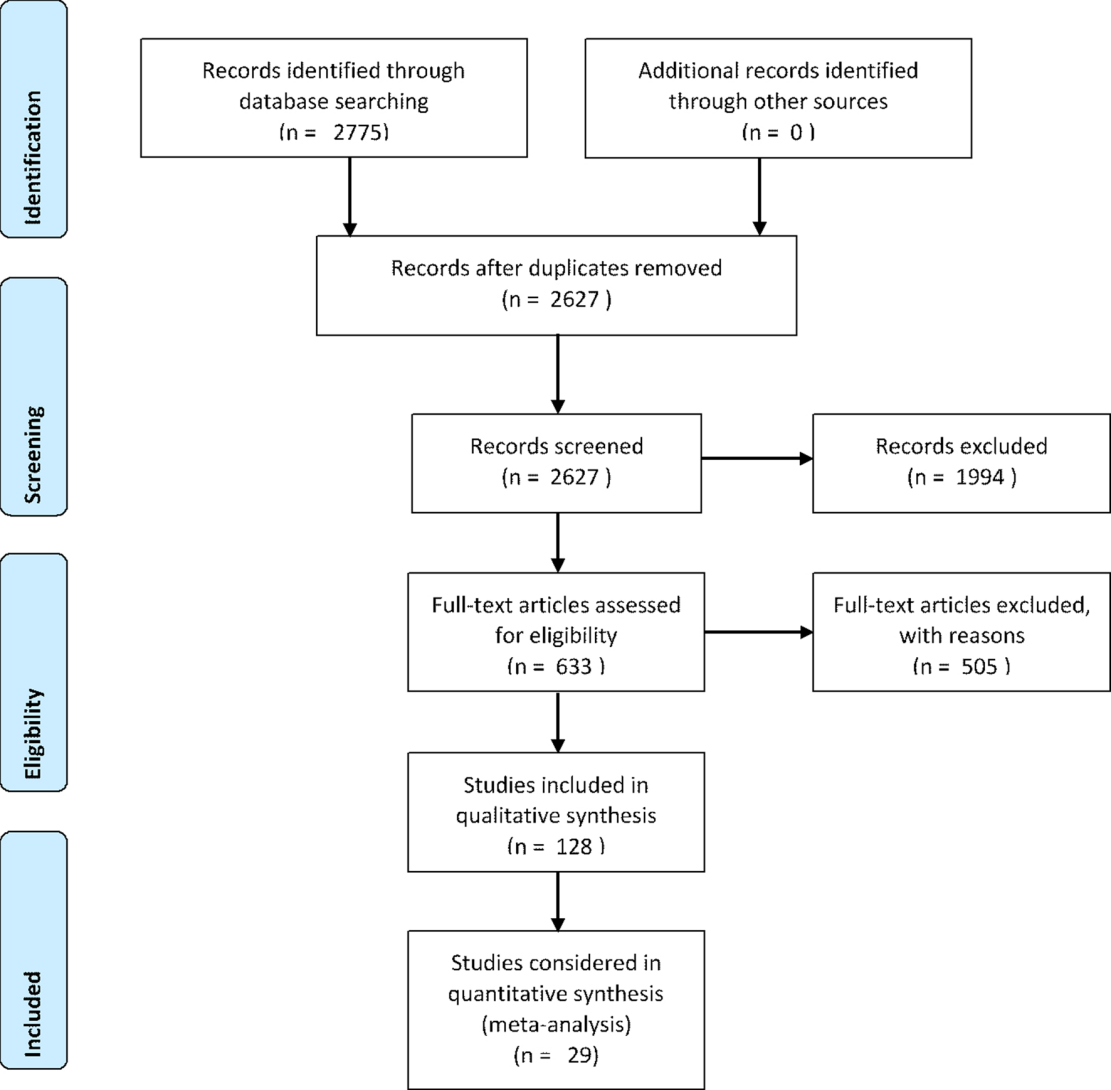
PRISMA flow chart detailing the literature search process.

A total of 2775 potential studies were identified through literature search , and 148 duplicates removed. Titles and abstracts for 2627 studies were screened. 633 studies fulfilled the inclusion criteria and were assessed for full-text evaluation. 505 full texts that did not meet the final inclusion criteria were excluded generating a final list of 128 studies included for systematic review analysis. The final number of studies included in the meta-analysis was 29 (Figure 1).

**Figure 2:**
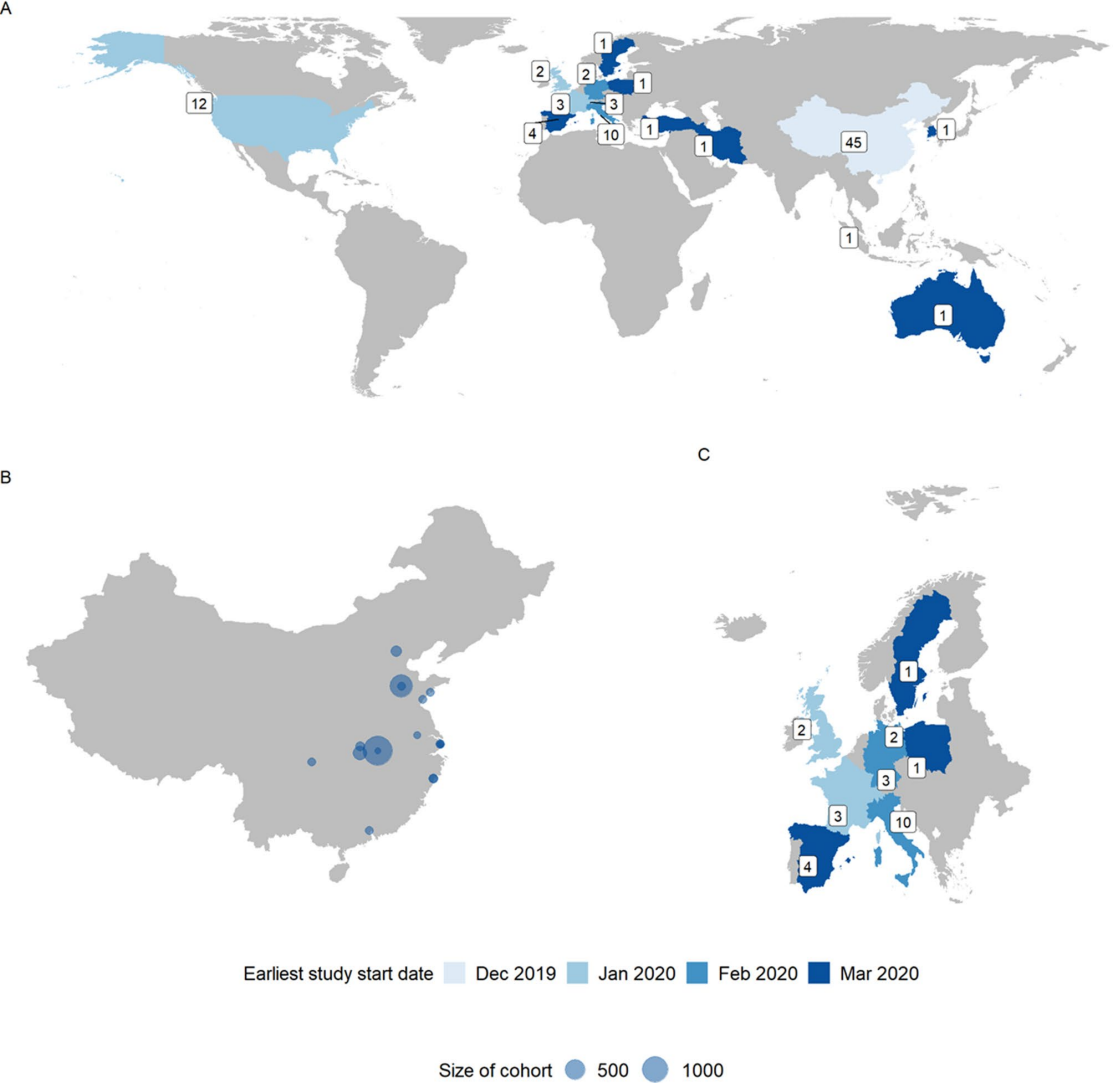
Distribution of studies over geography and time. (**A**) The distribution of studies globally where the label denotes number of studies in a country and colour denotes earliest recorded study start date. (**B**) Study locations in China where dot size corresponds to number of participants in entire study. (**C**) The distribution of studies in a subset of European countries where the label denotes number of studies in a country and colour denotes earliest recorded study start date. Studies including individuals from multiple countries, or without a city in China, were omitted from the map, n = 10.

The study locations and study dates followed the pandemic trajectory with the majority of early studies conducted in China (45 studies^11,17,25,26,31–69^) and later studies conducted in Europe (26 studies^10,72–94^) and North America (12 studies^97–106^) (Fig. 2). There were no studies that matched the inclusion criteria from South America or Africa. We include the study timings to show potential spatial and temporal bias in our data. Supplementary Table S1 details the studies included with indicators for the information extracted from each; the quality assessment scores for each study are presented in Supplementary Table S3.

### Susceptibility to infection

We identified only five studies^17,18,61,75,107^ that reported age-specific attack rates (AR) from contact tracing studies based on symptomatic surveillance for the index case and retrospective or prospective cohort studies, and these varied substantially between studies.

Based on contact-tracing studies in China, Bi et al.^17^, reported similar attack rates across all age groups in Shenzhen with a 7.4% attack rate in young children (< 10 years) compared to the population average of 6.6%. Liu et al.^61^ estimated a higher attack rate in Guangdong Province amongst children aged < 10 years and 10–19 years (5.7% and 4.0% respectively) compared to 20–29 year olds with the lowest AR of 2.3%. Conversely, PCR screening of 745 “highly suspected” children and 3174 adults found that adults were significantly more likely to test positive (1.3% in children vs 3.5% in adults)^107^. Those screened were individuals who had contact with a confirmed SARS-CoV-2 patient in the past 14 days or were identified as part of a familial outbreak in Guangzhou between 22 January and 20 February 2020.

**Figure 3:**
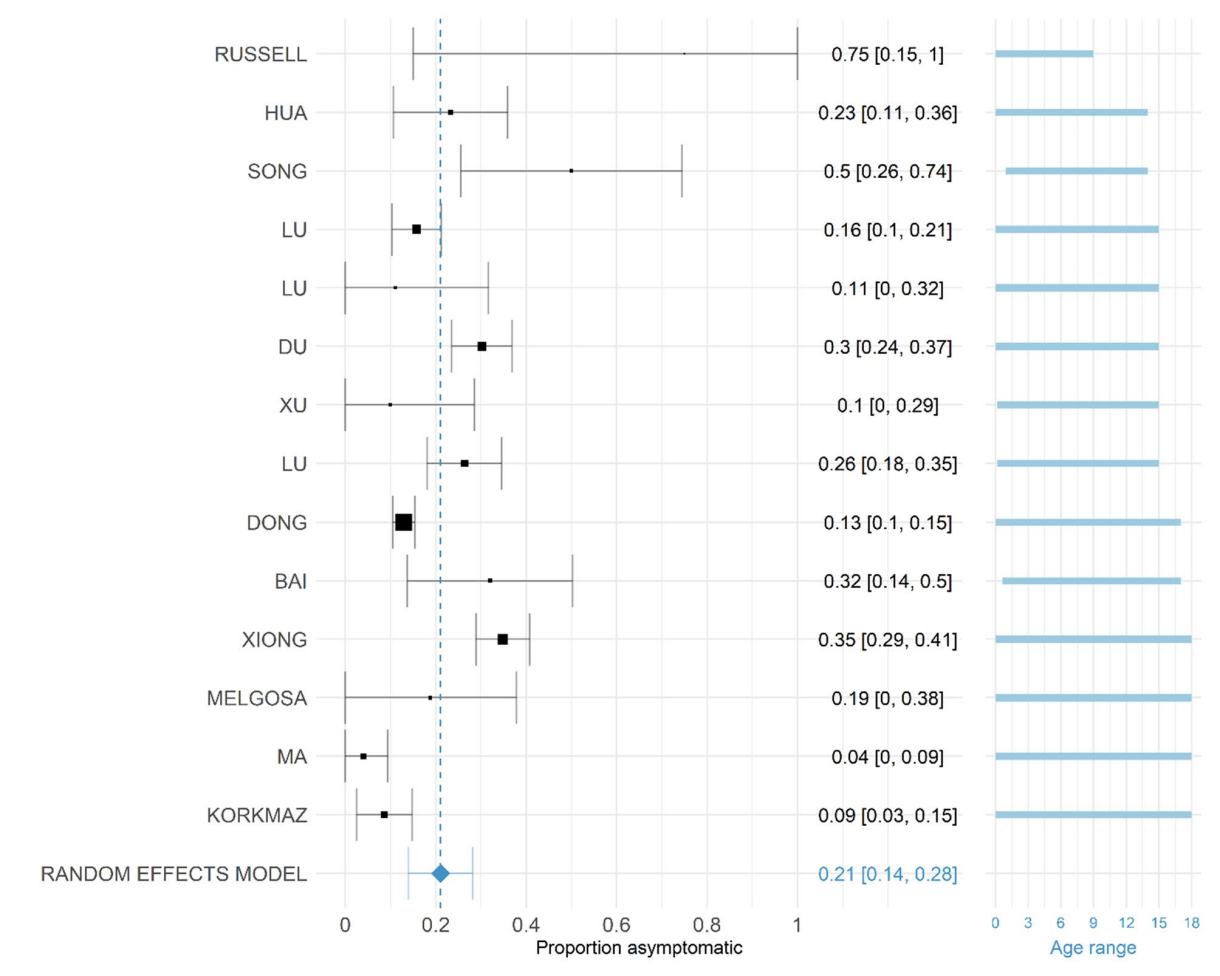
Proportion of SARS-CoV-2 positive children who are defined as asymptomatic at the time of the study in each published study. The random effects model result is given at the bottom indicated by a blue diamond. The squares are proportional in size to the number of COVID-19 positive individuals in the study. All studies were conducted in 2020. The labels on the left provide first author, the labels on the right give point estimate and confidence interval of the asymptomatic proportion estimated. Studies are ordered by the mean of the age range with age range given in blue on the right. Studies were included where recruitment criteria were clear and unbiased.

From studies in Europe, Lavezzo et al.^75^, did not detect a single SARS-CoV-2 positive amongst children aged < 10 years across two population wide surveys in Vo, Italy. They also found attack rates amongst older children aged 11–20 years were comparable to those observed in adult age groups. Similarly, population-based screening for SARS-CoV-2 in Iceland did not identify any SARS-CoV-2 positive children aged < 10 years. In individuals deemed at high risk due to recent overseas travel or COVID-like symptoms and targeted for testing, 6.7% of children aged < 10 years tested positive compared to 13.7% of those aged 10 years and above^18^. Prevalence by age group for relevant included studies is shown in Fig. 3.

We identified one study reporting age-specific seroprevalence from Geneva, Switzerland. Stringhini et al. found that young children aged 5–9 years had a significantly lower risk of being seropositive (RR 0.32, 95% CI 0.11–0.63) compared to adults 20–49 years^70^. This variability in age-specific infection rates between studies was also reflected amongst the studies that reported SARS-CoV-2 infection prevalence in children and adults (Fig. 5)^17,31,39,44,59,61,71,73,75,77,94,98,101,110–110^.

Although the source of infection amongst children could not be assessed systematically, studies identified through the review suggest that transmission to children tended to occur within household settings from other family members in family clusters, although for the majority schools were closed during the first wave^10,45^. Many children had a history of close contact with at least one parent who was SARS-CoV-2 positive^35,48,78^. Chen et al.^48^ observed, based on small numbers, that this family aggregation was consistent amongst infants and pre-school children (7 of 8), and school-aged children (14 of 16) but not in adolescents (1 of 8).

### Severity

#### Proportion asymptomatic

**Figure 4:**
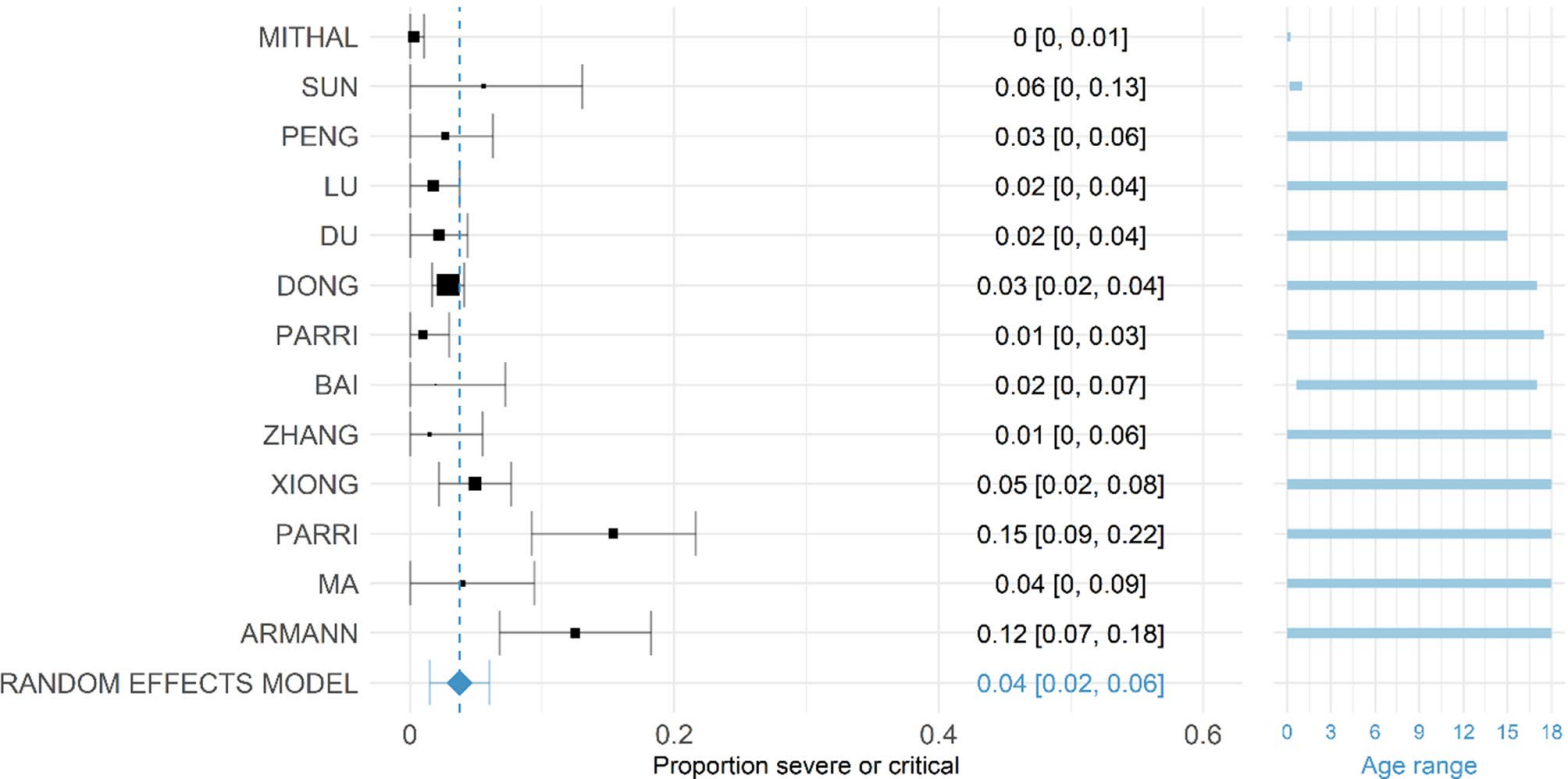
Proportion of COVID-19 positive children who were defined as severe or critical in each available study. The random effects model result is given at the bottom indicated by a blue diamond. The squares are proportional in size to the number of COVID-19 positive individuals in the study. All studies were conducted in 2020. The labels on the left provide first author, the labels on the right give point estimate and confidence interval of the proportion. Studies are ordered by the mean of the age range with age range given in blue on the right.

29 studies with information on the size of the population considered COVID-19 positive by PCR and the number of individuals considered asymptomatic in those aged 18 or less were included^10,33,41,42,45–47,49,50,54,55,58,62,64,65,114,67,76,77,79,83,84,87,90,100,107,108,111,112^. Most studies were cohort studies or case series; follow-up was sometimes not detailed. One study (Hu et al.^113^) was omitted during data extraction because the study population only selected asymptomatic children. The other studies were assessed for inclusion in the meta-analysis based on their recruitment of study participants and whether this was clear and unbiased. All studies are detailed in Table S4 in the supplementary material as well as a meta-analysis containing all studies. Fourteen studies fulfilled this additional criterion and the proportion of individuals considered asymptomatic in each included study is shown in Fig. 4. Our pooled estimate of asymptomatic children was 21.1% (95% CI: 14.0–28.1%) with a ${\mathrm{\tau }}^{2}$ (between-trial-variance) of 0.014 (95% CI: 0.004–0.057) and I^2^ statistic (percentage of variation due to heterogeneity, rather than chance) of 90.94% (95% CI: 77.44–97.70). This indicates low variance between studies, but that the variance is mostly due to heterogeneity rather than chance. Information on the size of the population considered COVID-19 positive by RT-PCR and the number of individuals considered asymptomatic in those aged 18 or less were included in our analysis. A funnel plot of these studies is given in Figure S2 in the Supplementary Material and shows reasonable symmetry, suggesting low publication bias; however, as mentioned previously, there is geographic and temporal bias in the studies included. It is also noted that there have been issues in assessing symmetry of funnel plots for other works^114,115^. These estimates are consistent with estimates of asymptomatic infections in the wider population (20%, 95% CI: 17–25%) although this was found to vary between settings^24^.

#### Proportion severe or critical

**Figure 5:**
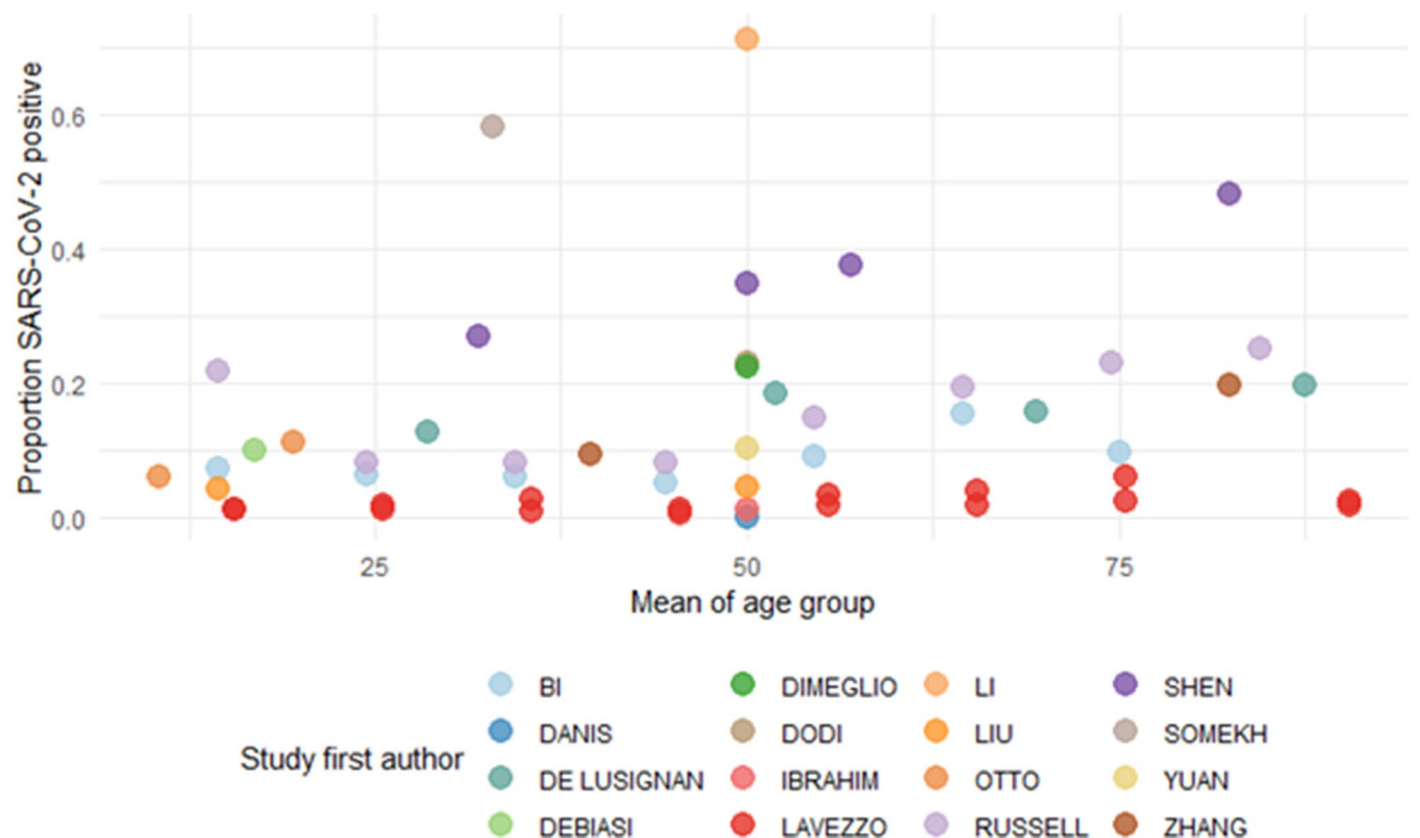
The age-specific prevalence shown as the proportion of confirmed SARS-CoV-2 cases by the mean age of the group. Studies were included if the maximum age was > 18 (i.e. they included both children and adults) and estimated the prevalence of infection in the cohort.

Thirteen studies with information on the size of the population considered COVID-19 positive by PCR and the number of individuals considered severe or critical in those aged 18 or younger were included^10,41,46,49,50,54,64,65,76,84,100,116,117^. Most studies were hospital-based and were based on PCR-confirmed cases only. Whilst the majority of studies were symptomatic children brought for clinical care, a small number of studies included asymptomatic PCR positive children at time of test. We again omitted Hu et al.^113^ as this study population focused on asymptomatic children only. We estimated that overall, 3.8% (95% CI: 1.5–6.0%) of children who were COVID-19 positive had severe or critical symptoms pooled across studies (Fig. 5). The estimated ${\tau }^{2}$ (between-trial-variance) was 0.0013 (95%CI: 0.0003–0.0049) and I^2^ of 91.30% (95% CI: 72.83–97.47), which indicates that most of the variability between studies is due to heterogeneity rather than chance, but that overall variance is small. A funnel plot of these studies is presented in Fig. S3 in the Supplementary Material showing reasonable symmetry.

### Transmissibility

We did not identify any studies that were designed specifically to measure SARS-CoV-2 transmissibility in children. As such, there is limited quantitative evidence to understand whether children are less likely to transmit SARS-CoV-2 to others compared to adults.

We identified one case series^52^ that confirmed SARS-CoV-2 transmission from an infant to both parents. Amongst paediatric cases identified in Ireland who attended school during their pre-symptomatic and symptomatic periods of infection (n = 3), no instances of onward transmission to either children or adults were identified^118^. Danis et al.^94^ detailed contact tracing of a cluster of cases in a French ski chalet. One paediatric case had a large number of contacts within a school setting (112 contacts) whilst symptomatic. However, contact tracing efforts did not identify any instances of onward transmission. Zachariah et al.^102^ reported two parents in New York, USA, developing symptoms consistent with COVID-19 whilst visiting their hospitalised child, although we note the parents could have been infected outside the hospital or by other hospitalised individuals. A large-scale contact tracing study in South Korea estimated that rates of infection were higher for contacts in households where the index patient was 10–19 years old (18.6%, 95% CI: 14.0–24.0%) compared to 11.8% (95% CI: 11.2–12.4%) of all household contacts of COVID-19 index cases^119^.

## Discussion

We identified 128 studies that provided information on the early impact of the COVID-19 pandemic on children and their potential role in transmission that met our eligibility criteria. Our study focused on studies published in the first eight months of the pandemic and represent knowledge and key information gaps early in the pandemic of a novel pathogen. Most studies were from China, Europe, and North America and there were no eligible studies from low- and middle-income countries notably from South America or Africa.

Although we were not able to estimate a pooled estimate for age-specific attack rates from the studies identified, they present a mixed picture of comparable, lower, or higher attack rates than adults depending on study setting^17,61^. Population-based PCR screening in Iceland^18^ and Italy^75^ and a seroprevalence survey in Geneva, Switzerland^70^ suggested that young children (5–9 years) were less susceptible to SARS-CoV-2 infection. A more recent hospital-based study in Italy found that children carried the virus less frequently than adults^120^. This agrees with a recent meta-analysis that included non-peer reviewed studies, which also estimated a lower susceptibility to infection in children and adolescents with a pooled odds ratio of 0.56 (95% CI: 0.37–0.85)^121,122^.Similarly a study carried out using household data in Bnei Brak in Israel found that children were less susceptible to SARS-CoV-2 infection that adults Other meta-analyses investigating data on the age of index cases and their contacts found no significant difference in susceptibility by age. However, when this was limited to household settings only, Thompson et al. found that adults were statistically significantly more susceptible to SARS-CoV-2 infection compared to children^123^. Jing et al. found similar age-dependent susceptibility in household settings^124^.

There is considerable variation in seroprevalence by age in studies published from multiple countries and settings. Across the USA, 8 out of 10 sites reported the lowest seroprevalence amongst individuals 18 years and younger. However, the sample sizes for children were small so it was difficult to accurately estimate seroprevalence in young children^125,126^. In Tokyo, Japan, no children recruited from two community clinics tested seropositive compared to an overall IgG seropositivity of 3.83% (95% CI: 2.76–5.16%)^127^. However, other studies have reported no differences in seropositivity between children and adults in Argentina^128^, Spain^129^, French Guiana^130^ , Germany^131^, and Italy^132^. Dingens et al.^133^ suggest that seroprevalence studies in children only are not able to quantify relative susceptibility compared to adults, but that the frequency of seropositivity is comparable to the overall incidence. Recent studies showing rapid waning of antibodies, particularly following mild symptoms which are more likely in children, may complicate the picture presented by seroprevalence surveys and their interpretation regarding susceptibility to infection^134,135^.

Adding to the body of evidence that SARS-CoV-2 infection in children are less severe than in adult populations, we estimated from 14 early studies that 21.1% (95% CI: 14.0–28.1%) of SARS-CoV-2 infections in children were asymptomatic. These studies were mainly paediatric cohort studies that swabbed children based on their recent exposure history with a confirmed COVID-19 patient. A small number of studies were retrospective or a case series, detailed in the Supplementary Material. Furthermore, our pooled estimate across 13 studies of the proportion of children with confirmed severe or critical COVID-19 symptoms was low at 3.8% (95% CI: 1.5–6.0%). Most of the 13 studies were based on symptomatic children brought to hospital for clinical care and only a small number of studies included asymptomatic PCR positive children at time of test. These estimates are consistent with recent findings from South Korea where 22.0% of SARS-CoV-2 positive children remained asymptomatic for the duration of infection and only 3% of cases were severe^136,137^. However, modelled estimates of the clinical fraction by age using data from 6 countries estimated that almost 80% of infections in children aged 10-19 years did not develop clinical symptoms i.e., were either asymptomatic or developed mild symptoms (only 21% (95% CrI: 12–31%) of infections in 10–19-year-olds leading to clinical symptoms)^11^. These estimates should be interpreted with caution as they rely on data from the early stages of the pandemic during which testing and reporting guidelines in countries were still evolving. The estimated proportion of asymptomatic infections in the population across all ages varies widely between studies. He et al., based on 41 studies of confirmed COVID-19 estimated a pooled percentage 15.6% (95% CI, 10.1–23.0%) infections being asymptomatic with significant heterogeneity noted among studies^138^. Another meta-analysis by Zhang et al.^139^ based on early data from the pandemic across all ages estimated pooled rates of intensive care unit admission, acute respiratory distress syndrome, and death of 10.9%, 18.4%, and 4.3% respectively.

It is important to note that this systematic review is based on studies conducted early in the pandemic, predominantly within China. Recent large-scale population-based studies run by the Office for National Statistics^140^ and REACT-1^141^ in the UK have suggested that the proportion of asymptomatic infections may be as high as 67% across all age groups in the UK. However, this figure also includes individuals who did not respond to questions related to symptoms and pre-symptomatic individuals for the ONS and REACT-1 studies, respectively.

As of November 29th 2020, children still represented less than 13% of overall COVID-19 cases reported to the European Centre for Disease Prevention and Control and deaths among cases under 18 years were extremely uncommon^3^. This is indicative that SARS-CoV-2 infections amongst children may be less symptomatic or severe than adults as testing policies across Europe thus far have prioritised symptomatic or hospitalised cases. Only a minority of children have required hospital care in the UK with only 1.5% (310 out of 20,133) of patients in the UK aged < 18 years^9^. These patterns were also observed in countries affected early on in the pandemic such as China and Italy with few child-hospitalisations^142^. Although we did not consider fatality estimates in this study, deaths are also highly age-dependent with low case and infection fatality estimates in children^8,143,144^.

There were limited studies that addressed the transmissibility of children but there was evidence of onward transmission from paediatric index cases^52^ and rates of COVID-19 being higher amongst household contacts of children in South Korea^119^. Contrastingly, detailed contact-tracing of secondary contacts of a child in France with a large number of contacts across 3 schools did not identify any secondary cases^94^. There were also no secondary cases reported in a school setting in Ireland^118^. Similarly, a seroprevalance study in Germany found no transmission from an index-person less 18 years to a household contact^131^. Milder symptoms in children may limit how much virus is expelled, given viral loads in children and adults may be comparable^145,146^, and therefore, how infectious children are. However, studies have shown that a substantial amount of transmission occurs before the onset of symptoms^24,26,147,148^, suggesting the relationship between transmissibility and symptoms is complex. A recent meta-analysis estimating secondary attack rates by age of the index case and their contact found no significant difference by age in transmissibility. When this was limited to household studies there was weak but not statistically significant evidence that children were less transmissible than adults^123^.

Overall, early evidence suggests that children are less likely to transmit SARS-CoV-2 compared to adults. A cluster-based study from Japan did not identify any children aged 0–19 years as a probable primary case of a cluster^149^, a national study in South Korea reported very low secondary household attack rates of 0.5% (95% CI: 0.0–2.6%) from paediatric index cases^150^, and a prospective study in New South Wales, Australia, similarly identified very few instances of onward transmission from a paediatric index case^151^. A detailed study from Tamil Nadu and Andhra Pradesh, India, based on data from 575,071 tested contacts of 84,965 confirmed cases found that the probability of transmission was assortative by age with the strongest effects observed in children aged 0–14 years and adults aged 65 years and above^152^.

Most countries implemented full or partial school-closures during the first wave of the pandemic. As schools and other educational institutions re-open across the world, school outbreaks have been increasingly reported, with a large outbreak resulting in a 13.2% attack rate in secondary school in Israel^153^, and 41 out of 825 primary, secondary and trade schools in Berlin reporting an outbreak of COVID-19 within 2 weeks of reopening^154–156^. However, it is difficult to determine whether transmission occurred primarily in schools or whether pupils were infected at home, or in other social settings. Nevertheless, high attack rates of 44% reported at a youth camp in Georgia, USA suggests that SARS-CoV-2 can transmit readily in young populations^157^. It is likely that school outbreaks will mirror increasing prevalence in the community itself^158^. Whether schools themselves are the drivers of transmission is still unknown, particularly with many containment measures such as cohorts implemented since reopening.

There are a number of limitations to our study. By the end of the review period (July 2020), there were no studies from Africa or South America that met the inclusion criteria. Contact patterns and household structures may differ substantially in these settings from the predominantly high-income countries considered here, which may result in differences that we have not captured. Although we have estimated the pooled proportion asymptomatic based on the values reported by the study authors, we could not assess whether individuals went on to develop symptoms as very few studies reported the length of follow-up, or loss to follow-up. As such, the definition of asymptomatic may vary between included studies, affecting the estimate. Most of the studies considered were undertaken when non-pharmaceutical interventions, including school closures, case isolation, workplace closures and other social distancing measures, were implemented. The substantial change in contact patterns and the increased mixing within households may mean that children have had fewer opportunities for contact and this decreased the chances of becoming infected or transmitting the virus. Therefore, contact tracing studies have likely disproportionately identified transmission within the household^39^. As restrictions are lifted, we may expect to see a larger role of children in transmission as contact rates will significantly increase. For both population-based infection surveys and seroprevalence studies, the number of children sampled was small and very few studies differentiated between young children and adolescents who may have different risk profiles. Finally, children may be more likely to be tested depending on healthcare seeking behaviour of parents or because in general they may be more prone to illness causing influenza-like illness symptoms. Both factors may contribute in a higher asymptomatic proportion. This review considers only those studies published early in the pandemic. The newly emerging strains of SARS-CoV-2 may exhibit different properties to the earlier circulating strains, such as higher transmissibility in children or changes in severity profiles^159^, which warrant further investigation.

## Conclusions

With many countries across the world now in their second or third wave of the pandemic, there is increasing evidence that children are susceptible to SARS-CoV-2 infection, although perhaps to a lesser extent than adults. It is clear, however, that many children experience clinically mild disease or remain asymptomatically infected. Although severe disease in children does occur, fatalities due to COVID-19 remain rare. Whilst there is evidence to suggest that children are capable of transmitting SARS-CoV-2, more comprehensive contact-tracing studies combined with serosurveys are needed to quantify their transmissibility relative to adults and determine whether they contribute significantly to the outbreaks. Studies showing school reopening can address these data gaps and have begun to show the role of children in the pandemic, although conclusions around the role of children in transmission are still mixed^153,160–163^. It is critical that testing regimes and study protocols are in place that will allow us to better understand the role of children in this pandemic.

## Supplementary Information

Supplementary Information 1.

Supplementary Information 2.
