# Publisher Correction: Children’s role in the COVID-19 pandemic: a systematic review of early surveillance data on susceptibility, severity, and transmissibility

**DOI:** 10.1038/s41598-021-97183-w

**Published:** 2021-09-16

**Authors:** Katy A. M. Gaythorpe, Sangeeta Bhatia, Tara Mangal, H. Juliette T. Unwin, Natsuko Imai, Gina Cuomo-Dannenburg, Caroline E. Walters, Elita Jauneikaite, Helena Bayley, Mara D. Kont, Andria Mousa, Lilith K. Whittles, Steven Riley, Neil M. Ferguson

**Affiliations:** 1 MRC Centre for Global Infectious Disease Analysis and WHO Collaborating Centre for Infectious Disease Modelling, Abdul Latif Jameel Institute for Disease and Emergency Analytics, Imperial College London, London, UK; 2 Department of Physics, University of Oxford, Oxford, UK

Correction to: *Scientific Reports* 10.1038/s41598-021-92500-9, published online 06 July 2021

The original version of this Article contained errors.

The legends of Figure 3, Figure 4 and Figure 5 were inadvertently switched.

The legend of Figure 3:

“The age-specific prevalence shown as the proportion of confirmed SARS-CoV-2 cases by the mean age of the group. Studies were included if the maximum age was > 18 (i.e. they included both children and adults) and estimated the prevalence of infection in the cohort.”

now reads:

“Proportion of SARS-CoV-2 positive children who are defined as asymptomatic at the time of the study in each published study. The random effects model result is given at the bottom indicated by a blue diamond. The squares are proportional in size to the number of COVID-19 positive individuals in the study. All studies were conducted in 2020. The labels on the left provide first author, the labels on the right give point estimate and confidence interval of the asymptomatic proportion estimated. Studies are ordered by the mean of the age range with age range given in blue on the right. Studies were included where recruitment criteria were clear and unbiased.”

The legend of Figure 4:

“Proportion of SARS-CoV-2 positive children who are defined as asymptomatic at the time of the study in each published study. The random effects model result is given at the bottom indicated by a blue diamond. The squares are proportional in size to the number of COVID-19 positive individuals in the study. All studies were conducted in 2020. The labels on the left provide first author, the labels on the right give point estimate and confidence interval of the asymptomatic proportion estimated. Studies are ordered by the mean of the age range with age range given in blue on the right. Studies were included where recruitment criteria were clear and unbiased.”

now reads:

“Proportion of COVID-19 positive children who were defined as severe or critical in each available study. The random effects model result is given at the bottom indicated by a blue diamond. The squares are proportional in size to the number of COVID-19 positive individuals in the study. All studies were conducted in 2020. The labels on the left provide first author, the labels on the right give point estimate and confidence interval of the proportion. Studies are ordered by the mean of the age range with age range given in blue on the right.”

The legend of Figure 5:

“Proportion of COVID-19 positive children who were defined as severe or critical in each available study. The random effects model result is given at the bottom indicated by a blue diamond. The squares are proportional in size to the number of COVID-19 positive individuals in the study. All studies were conducted in 2020. The labels on the left provide first author, the labels on the right give point estimate and confidence interval of the proportion. Studies are ordered by the mean of the age range with age range given in blue on the right.”

now reads:

“The age-specific prevalence shown as the proportion of confirmed SARS-CoV-2 cases by the mean age of the group. Studies were included if the maximum age was > 18 (i.e. they included both children and adults) and estimated the prevalence of infection in the cohort.”

Additionally, Reference 132 was incorrectly listed in the Reference List as Reference 158. As a result, the references have been renumbered.

Furthermore, a reference was omitted at the end of the following sentence. It now reads,

“As schools and other educational institutions re-open across the world, school outbreaks have been increasingly reported, with a large outbreak resulting in a 13.2% attack rate in secondary school in Israel^153^, and 41 out of 825 primary, secondary and trade schools in Berlin reporting an outbreak of COVID-19 within 2 weeks of reopening^154,155,156^.”

The original Article has been corrected.

