# Supplementary material for: Children’s role in the COVID-19 pandemic: a systematic review of early surveillance data on susceptibility, severity, and transmissibility
Source: Sci Rep. 2021 Jul 6;11:13903. doi: 10.1038/s41598-021-92500-9 (PMC8260804; doi:10.1038/s41598-021-92500-9)
Supplement: Supplementary file 1 — Supplementary Information 1. [file 41598_2021_92500_MOESM1_ESM.docx]

**Supplementary Material:** **Children’s role in the COVID-19 pandemic: a systematic review of early surveillance data on susceptibility, severity, and transmissibility**

Katy A.M. Gaythorpe^1*^, Sangeeta Bhatia^1*^, Tara Mangal^1*^, H. Juliette T. Unwin^1*^, Natsuko Imai^1*^, Gina Cuomo-Dannenburg^1^^, Caroline E. Walters^1^^, Elita Jauneikaite^1^^, Helena Bayley^2^, Mara D. Kont^1^, Andria Mousa^1^, Lilith Whittles^1^, Steven Riley^1^, Neil M. Ferguson^1^

^1^ MRC Centre for Global Infectious Disease Analysis & WHO Collaborating Centre for Infectious Disease Modelling, Abdul Latif Jameel Institute for Disease and Emergency Analytics, Imperial College London

^2^Department of Physics, University of Oxford

Correspondence: [k.gaythorpe@imperial.ac.uk](mailto:k.gaythorpe@imperial.ac.uk) ,

*contributed equally; ^contributed equally,

## Section S1: Inclusion and Exclusion criteria

Our inclusion and exclusion criteria are as follows:

Inclusion criteria:

Mention of severity in children

- Mention of incidence in children
- Estimates of population-level incidence / attack rates
- Mention of infectiousness or secondary attack rate from infected children
- Mention of onwards transmission from children
- Mentions of asymptomatic infection in children

Exclusion criteria:

- Non-English language articles
- Not peer reviewed articles
- Duplicated articles (with the exclusion of pre-prints where the duplicate is the peer reviewed version)
- SARS-CoV-2 not included or the focus of the paper
- Articles covering study periods before December 2019
- Studies that excluded children and adolescents from their participants.
- No mention of severity in children OR incidence/prevalence in children OR infectiousness of children OR clusters of transmission OR vertical transmission from mother-to-child
- Editorials/commentaries/opinion/news articles that present no new data/research
- Modelling studies that are not age structured
- Modelling studies that are not fitted to real data

## Section S2: Supplementary Tables

*Supplementary Table S1: Table of papers included in our study with indicators for the information extracted from each study.*

| Study | Susceptibility | Transmissibility | Severity |
| --- | --- | --- | --- |
| Armann 2020 [1] | x |  |  |
| Asfahan 2020 [2] |  |  | x |
| Bai 2020 [3] |  |  | x |
| Belhadjer 2020 [4] |  |  | x |
| Belot 2020 [5] |  |  | x |
| Bi 2020 [6] | x |  | x |
| Brambilla 2020 [7] | x |  | x |
| Buonsenso 2020 [8] | x |  | x |
| Cai 2020 [9] | x | x | x |
| Chen 2020 [10] |  |  | x |
| Chen 2020 [11] | x | x |  |
| Danis 2020 [12] | x | x | x |
| DeBiasi 2020 [13] | x |  | x |
| deCeano-Vivas 2020 [14] | x |  | x |
| DeIoris 2020 [15] |  | x | x |
| deLusignan 2020 [16] | x |  |  |
| Dimeglio 2020 [17] | x |  | x |
| Docherty 2020 [18] | x |  | x |
| Dodi 2020 [19] | x | x | x |
| Dong 2020 [20] | x |  | x |
| Dong 2020 [21] |  |  | x |
| Du 2020 [22] |  |  | x |
| Du 2020 [23] |  | x | x |
| Du 2020 [24] | x |  | x |
| Dufort 2020 [25] |  |  | x |
| Foster 2020 [26] | x |  |  |
| Gao 2020 [27] |  | x |  |
| Garazzino 2020 [28] | x | x | x |
| García-Salido 2020 [29] |  |  |  |
| González Cortés 2020 [30] |  |  | x |
| Götzinger 2020 [31] |  |  | x |
| Guan 2020 [32] | x |  | x |
| Gudbjartsson 2020 [33] | x |  |  |
| Gujski 2020 [34] | x |  |  |
| Han 2020 [35] |  | x | x |
| Han 2020 [36] |  |  | x |
| Harman 2020 [37] |  |  | x |
| He 2020 [38] | x |  | x |
| Heavey 2020 [39] |  | x |  |
| Hildenwall 2020 [40] | x | x | x |
| Hrusak 2020 [41] |  |  |  |
| Hu 2020 [42] |  |  | x |
| Hua 2020 [43] |  | x | x |
| Ibrahim 2020 [44] | x |  |  |
| Ji 2020 [45] | x |  | x |
| Korean Society of Infectious Diseases 2020 [46] | x |  |  |
| Korkmaz 2020 [47] |  | x | x |
| L'Huillier 2020 [48] |  | x | x |
| Lavezzo 2020 [49] | x |  |  |
| Li 2020 [50] | x |  |  |
| Li 2020 [51] |  |  | x |
| Li 2020 [52] | x |  | x |
| Liu 2020 [53] | x |  | x |
| Liu 2020 [54] | x |  | x |
| Liu 2020 [55] | x |  |  |
| Liu 2020 [56] | x | x | x |
| Lu 2020 [57] | x |  | x |
| Lu 2020 [58] | x | x | x |
| Lu 2020 [59] |  | x | x |
| Ma 2020 [60] | x |  | x |
| Ma 2020 [61] |  |  | x |
| Mannheim 2020 [62] | x | x | x |
| Marlais 2020 [63] | x |  | x |
| Martínez-Perez 2020 [64] |  |  | x |
| Melgosa 2020 [65] | x | x | x |
| Mithal 2020 [66] | x | x | x |
| Mizumoto 2020 [67] | x |  |  |
| Moratto 2020 [68] | x |  | x |
| Ng 2020 [69] | x |  | x |
| Otto 2020 [70] | x |  |  |
| Oualha 2020 [71] |  | x | x |
| Pan 2020 [72] | x |  | x |
| Park 2020 [73] |  | x |  |
| Parri 2020 [74] | x |  | x |
| Parri 2020 [75] | x | x | x |
| Pathak 2020 [76] |  |  | x |
| Peng 2020 [77] | x | x | x |
| Posfay-Barbe 2020 [78] | x | x | x |
| Pouletty 2020 [79] |  | x | x |
| Preßler 2020 [80] | x | x | x |
| Qiu 2020 [81] | x |  | x |
| Qiu 2020 [82] |  |  | x |
| Ranabothu 2020 [83] | x |  | x |
| Rezaei 2020 [84] | x |  | x |
| Rha 2020 [85] | x |  | x |
| Russell 2020 [86] | x |  |  |
| Schwierzeck 2020 [87] | x |  |  |
| Shekerdemian 2020 [88] | x |  | x |
| Shen 2020 [89] | x |  |  |
| Shi 2020 [90] | x | x |  |
| Soltani 2020 [91] |  |  | x |
| Somekh 2020 [92] |  | x |  |
| Son 2020 [93] |  |  | x |
| Song 2020 [94] | x | x | x |
| Song 2020 [95] |  |  | x |
| Spiteri 2020 [96] | x |  |  |
| Stringhini 2020 [97] | x |  |  |
| Sun 2020 [98] | x |  |  |
| Sun 2020 [99] |  |  | x |
| Talarico 2020 [100] | x |  |  |
| Tan 2020 [101] | x | x | x |
| Tian 2020 [102] | x |  | x |
| Tong 2020 [103] | x |  | x |
| Turner 2020 [104] | x |  |  |
| Valente 2020 [105] |  | x | x |
| Verdoni 2020 [106] |  |  | x |
| Wu 2020 [107] | x |  | x |
| Wu 2020 [108] | x | x | x |
| Wu 2020 [109] |  |  | x |
| Xia 2020 [110] | x |  | x |
| Xiong 2020 [111] |  |  | x |
| Xiong 2020 [112] |  | x | x |
| Xu 2020 [113] |  | x | x |
| Xu 2020 [114] | x | x |  |
| Xu 2020 [115] | x |  |  |
| Ye 2020 [116] | x |  | x |
| Yongchen 2020 [117] |  |  | x |
| Yuan 2020 [118] | x |  |  |
| Yung 2020 [119] |  | x |  |
| Zachariah 2020 [120] | x | x | x |
| Zachariah 2020 [121] | x | x | x |
| Zhang 2020 [122] | x |  |  |
| Zhang 2020 [123] | x |  |  |
| Zhang 2020 [124] |  | x | x |
| Zhang 2020 [125] |  | x |  |
| Zhao 2020 [126] | x |  |  |
| Zhao 2020 [127] |  |  | x |
| Zheng 2020 [128] | x | x | x |

*Supplementary Table S2: Questions used to do quality assessment for the included publications for the review.*

| Item | Criteria | Notes |
| --- | --- | --- |
| 1 | Was the study question or objective clearly stated? | score 1 if 'yes', 0.5 if partially stated, 0 if 'no' or unclear |
| 2 | Was the study setting (geography) and period (time) clearly described? | score 1 if 'yes', 0.5 if partially described, 0 if 'no' or unclear |
| 3 | Was the sampling frame a true or close representation of the target population? | score 1 if 'yes', 0.5 if partially stated, 0 if 'no' or unclear |
| 4 | Were case definitions clearly and fully described? (eg. asymptomatic cases, mild, moderate, severe cases) | score 1 if 'yes', 0.5 if partially described, 0 if 'no' or unclear |
| 5 | Is the testing procedure clearly mentioned and appropriate for the study? Do we know how many patients were given antigen tests/PCR/clinically identified | score 1 if 'yes', 0.5 if partially stated, 0 if 'no' or unclear |
| 6 | Are the eligibility criteria (inclusion and exclusion criteria) for entry into the study explicit and appropriate? | score 1 if 'yes', 0 if 'no' or unclear |
| 7 | Was the length of follow-up reported and loss to follow-up if appropriate? | score 1 if 'yes', 0.5 if partially stated, 0 if 'no' or unclear |
| 8 | Were the outcomes reported i.e. were the number of deaths/recoveries reported? | score 1 if 'yes', 0.5 if partially stated, 0 if 'no' or unclear |
| 9 | Are the characteristics of the participants included in the study described? Age breakdown - if very broad(e.g. <=17), give low score | score 1 if patient characteristics reported individually, 0.5 if summarized as a group, 0 if not described |
| 10 | Are the conclusions of the study supported by results? | score 1 if 'yes', 0.5 if partially stated, 0 if 'no' or unclear |
|  |  |  |
|  |  |  |
|  |  |  |
| Good | Score ≥7 |  |
| Fair | Score 5 - 6 |  |
| Poor | Score <5 |  |

*Supplementary Table S3: Quality assessment scores*

| No. | Study | QAs questions | | | | | | | | | | Score | Rating |
| --- | --- | --- | --- | --- | --- | --- | --- | --- | --- | --- | --- | --- | --- |
|  |  | 1 | 2 | 3 | 4 | 5 | 6 | 7 | 8 | 9 | 10 |  |  |
| 1 | Bi *et al*, 2020 | 1 | 1 | 1 | 1 | 1 | 1 | 0.5 | 1 | 1 | 1 | 9.5 | Good |
| 2 | Lu *et al,* 2020 | 0.5 | 1 | 1 | 0.5 | 1 | 1 | 0 | 1 | 1 | 1 | 8 | Good |
| 3 | Hu *et al,* 2020 | 1 | 1 | 0 | 0 | 1 | 0.5 | 0 | 1 | 1 | 1 | 6.5 | Fair |
| 4 | Cai *et al*, 2020 | 0.5 | 1 | 1 | 1 | 1 | 1 | 1 | 1 | 1 | 1 | 9.5 | Good |
| 5 | Chen *et* *al*, 2020 | 1 | 1 | 1 | 0.5 | 1 | 1 | 0.5 | 1 | 0 | 1 | 8 | Good |
| 6 | Kostas et al, 2020 | 1 | 1 | 0.5 | 1 | 1 | 1 | 1 | 1 | 0.5 | 1 | 9 | Good |
| 7 | Dong et al, 2020 | 1 | 1 | 0 | 1 | 1 | 0 | 1 | 1 | 1 | 1 | 8 | Good |
| 8 | Du et al, 2020 | 1 | 1 | 1 | 1 | 1 | 0.5 | 0 | 1 | 0 | 1 | 7.5 | Good |
| 9 | Dong et al, 2020 | 1 | 0.5 | 1 | 1 | 1 | 1 | 0 | 0 | 1 | 1 | 7.5 | Good |
| 10 | CDC Covid-response team | 0 | 1 | 1 | 0 | 0.5 | 0.5 | 0 | 0.5 | 0.5 | 1 | 5 | Fair |
| 11 | Guan et al, 2020 | 1 | 1 | 1 | 0.5 | 1 | 0.5 | 1 | 1 | 0.5 | 1 | 8.5 | Good |
| 12 | Gudbjrtsson et al, 2020 | 1 | 0.5 | 1 | 0 | 1 | 0.5 | 0 | 0 | 0 | 1 | 5 | Poor |
| 13 | Sun et al, 2020 | 0.5 | 0 | 0 | 0 | 0 | 0 | 0 | 0.5 | 0 | 1 | 2 | Poor |
| 14 | Spiteri et al, 2020 | 1 | 0.5 | 1 | 0.5 | 1 | 0 | 0.5 | 1 | 0.5 | 1 | 7 | Good |
| 15 | Schwierzeck et al, 2020 | 1 | 0.5 | 0 | 0.5 | 0.5 | 0.5 | 0.5 | 0 | 0.5 | 1 | 5 | Fair |
| 16 | He et al, 2020 | 0.5 | 0.5 | 0 | 0.5 | 0.5 | 0 | 0 | 0 | 0 | 0.5 | 2.5 | Poor |
| 17 | Ji et al, 2020 | 1 | 0.5 | 1 | 1 | 1 | 0.5 | 0 | 0.5 | 0.5 | 1 | 7 | Good |
| 18 | Xu et al, 2020 | 1 | 1 | 0.5 | 0.5 | 0.5 | 0.5 | 0 | 0 | 1 | 0.5 | 5.5 | Fair |
| 19 | Lavezzo et al, 2020 | 1 | 1 | 1 | 1 | 1 | 1 | 0.5 | 0 | 0.5 | 1 | 8 | Good |
| 20 | Li et al, 2020 | 1 | 1 | 0 | 1 | 1 | 1 | 0 | 1 | 0 | 0.5 | 6.5 | Fair |
| 21 | Xu et al, 2020 | 1 | 1 | 1 | 0 | 1 | 0.5 | 0.5 | 0 | 0.5 | 1 | 6.5 | Fair |
| 22 | Parri et al, 2020 | 1 | 1 | 0.5 | 1 | 1 | 0.5 | 0 | 1 | 1 | 1 | 8 | Good |
| 23 | Song et al, 2020 | 1 | 1 | 0.5 | 1 | 1 | 0.5 | 1 | 1 | 1 | 1 | 9 | Good |
| 24 | Song et al, 2020 | 1 | 1 | 0.5 | 0 | 1 | 0.5 | 0 | 1 | 1 | 1 | 7 | Good |
| 25 | Xia et al, 2020 | 1 | 1 | 0.5 | 0 | 0.5 | 0.5 | 0.5 | 1 | 0.5 | 1 | 6.5 | Fair |
| 26 | Shi et al, 2020 | 1 | 1 | 1 | 0 | 1 | 1 | 0.5 | 0 | 0.5 | 1 | 7 | Good |
| 27 | Russell et al, 2020 | 1 | 1 | 0.5 | 1 | 0.5 | 1 | 1 | 1 | 1 | 1 | 9 | Good |
| 28 | Qiu et al, 2020 | 1 | 1 | 1 | 1 | 1 | 1 | 1 | 1 | 0.5 | 1 | 9.5 | Good |
| 29 | Melgosa et al, 2020 | 1 | 1 | 0.5 | 0.5 | 0.5 | 0 | 1 | 1 | 0.5 | 1 | 7 | Good |
| 30 | Dodi et al, 2020 | 0 | 1 | 1 | 1 | 1 | 0.5 | 1 | 0 | 1 | 1 | 7.5 | Good |
| 31 | Gujski et al, 2020 | 1 | 1 | 1 | 0 | 1 | 0.5 | 1 | 1 | 0.5 | 1 | 8 | Good |
| 32 | Rha et al, 2020 | 1 | 1 | 1 | 0.5 | 1 | 1 | 1 | 1 | 0 | 1 | 8.5 | Good |
| 33 | Ranabothu et al, 2020 | 0.5 | 0.5 | 1 | 0.5 | 0.5 | 1 | 0 | 1 | 1 | 0.5 | 6.5 | Fair |
| 34 | Mithal et al, 2020 | 1 | 1 | 1 | 0 | 1 | 1 | 0.5 | 0.5 | 0.5 | 1 | 7.5 | Good |
| 35 | Garazzino et al, 2020 | 1 | 0.5 | 1 | 0.5 | 1 | 1 | 0.5 | 1 | 1 | 1 | 8.5 | Good |
| 36 | Stringhini et al, 2020 | 1 | 1 | 1 | 0 | 1 | 1 | 0 | 0 | 0.5 | 1 | 6.5 | Fair |
| 37 | Otto et al, 2020 | 1 | 1 | 0.5 | 0.5 | 1 | 1 | 0.5 | 1 | 0.5 | 1 | 8 | Good |
| 38 | Wu et al, 2020 | 1 | 1 | 0.5 | 0.5 | 0.5 | 0.5 | 1 | 1 | 0 | 1 | 7 | Good |
| 39 | Zheng et al, 2020 | 1 | 1 | 0.5 | 0.5 | 0.5 | 0.5 | 0.5 | 1 | 0.5 | 1 | 7 | Good |
| 40 | Götzinger et al, 2020 | 1 | 1 | 1 | 0.5 | 1 | 1 | 1 | 1 | 1 | 1 | 9.5 | Good |
| 41 | Zhang et al, 2020 | 1 | 1 | 1 | 0 | 0 | 0.5 | 0 | 0 | 0.5 | 1 | 5 | Poor |
| 42 | Xiong et al, 2020 | 1 | 1 | 0 | 0.5 | 0.5 | 0 | 0 | 0 | 0.5 | 1 | 4.5 | Poor |
| 43 | L’Huillier et al, 2020 | 0.5 | 1 | 0 | 0 | 1 | 0 | 0 | 0 | 0.5 | 1 | 4 | Poor |
| 44 | Hua et al, 2020 | 1 | 1 | 1 | 0.5 | 1 | 0 | 1 | 1 | 0.5 | 1 | 8 | Good |
| 45 | DeIoris et al, 2020 | 1 | 1 | 0.5 | 1 | 1 | 0.5 | 1 | 1 | 1 | 1 | 9 | Good |
| 46 | Hildenwall et al, 2020 | 0.5 | 1 | 1 | 0.5 | 1 | 1 | 0.5 | 1 | 0.5 | 1 | 8 | Good |
| 47 | Zachariah et al, 2020 | 1 | 1 | 0 | 0.5 | 1 | 1 | 0 | 0.5 | 0 | 1 | 6 | Fair |
| 48 | Dimeglio et al, 2020 | 0 | 1 | 1 | 0 | 1 | 1 | 0 | 1 | 0 | 0.5 | 5.5 | Fair |
| 49 | Moratto et al, 2020 | 0.5 | 1 | 0 | 0.5 | 0.5 | 0.5 | 0 | 0 | 1 | 1 | 5 | Fair |
| 50 | Brambilla et al, 2020 | 1 | 1 | 1 | 1 | 1 | 1 | 0 | 1 | 0 | 1 | 8 | Good |
| 51 | Yung et al, 2020 | 1 | 0.5 | 0 | 0.5 | 1 | 1 | 1 | 1 | 0.5 | 1 | 7.5 | Good |
| 52 | Docherty et al, 2020 | 1 | 1 | 1 | 0 | 0.5 | 1 | 1 | 1 | 0.5 | 1 | 8 | Good |
| 53 | Li et al, 2020 | 1 | 1 | 0.5 | 0 | 1 | 1 | 0 | 0 | 0.5 | 1 | 6 | Fair |
| 54 | Ibrahim et al, 2020 | 1 | 1 | 0.5 | 0 | 1 | 0.5 | 0 | 0 | 0.5 | 1 | 5.5 | Fair |
| 55 | Yuan et al, 2020 | 1 | 1 | 1 | 0 | 1 | 0.5 | 0 | 0 | 0.5 | 1 | 6 | Fair |
| 56 | Wu et al, 2020 | 1 | 1 | 0 | 1 | 0.5 | 0 | 1 | 1 | 0.5 | 1 | 7 | Good |
| 57 | Mannheim et al, 2020 | 1 | 1 | 1 | 0.5 | 1 | 0.5 | 1 | 1 | 0.5 | 1 | 8.5 | Good |
| 58 | Valente et al, 2020 | 1 | 1 | 0 | 0.5 | 0.5 | 0.5 | 0.5 | 1 | 1 | 1 | 7 | Good |
| 59 | Parri et al, 2020 | 1 | 1 | 1 | 1 | 0.5 | 1 | 0 | 1 | 0.5 | 1 | 8 | Good |
| 60 | Belhadjer et al, 2020 | 1 | 1 | 1 | 0.5 | 1 | 1 | 1 | 1 | 0 | 0.5 | 8 | Good |
| 61 | deCeano-Vivas et al, 2020 | 1 | 1 | 1 | 0 | 1 | 1 | 0 | 1 | 0.5 | 1 | 7.5 | Good |
| 62 | Du et al, 2020 | 1 | 1 | 0.5 | 1 | 1 | 0.5 | 1 | 1 | 1 | 1 | 9 | Good |
| 63 | Marlais et al, 2020 | 0.5 | 1 | 1 | 0 | 0 | 1 | 0 | 0.5 | 0.5 | 0.5 | 5 | Fair |
| 64 | deLusignan et al, 2020 | 1 | 1 | 1 | 1 | 1 | 1 | 1 | 1 | 0 | 1 | 9 | Good |
| 65 | GonzálezCortés et al, 2020 | 1 | 1 | 1 | 0.5 | 0 | 0 | 0 | 0 | 0 | 1 | 4.5 | Poor |
| 66 | Sun et al, 2020 | 1 | 1 | 0.5 | 0.5 | 0.5 | 0 | 1 | 1 | 0.5 | 1 | 7 | Good |
| 67 | Lu et al, 2020 | 1 | 1 | 0.5 | 0 | 1 | 0.5 | 0 | 1 | 0.5 | 1 | 6.5 | Fair |
| 68 | Foster et al, 2020 | 1 | 1 | 1 | 0.5 | 1 | 1 | 0 | 1 | 0 | 1 | 7.5 | Good |
| 69 | Armann et al, 2020 | 0.5 | 1 | 0.5 | 0.5 | 0.5 | 1 | 0.5 | 1 | 0 | 1 | 6.5 | Fair |
| 70 | Chen et al, 2020 | 1 | 0.5 | 0 | 0 | 1 | 0 | 0 | 1 | 1 | 1 | 5.5 | Fair |
| 71 | Du et al, 2020 | 1 | 1 | 0.5 | 1 | 0.5 | 1 | 1 | 1 | 0.5 | 1 | 8.5 | Good |
| 72 | Harman et al, 2020 | 1 | 1 | 0 | 0.5 | 0.5 | 0.5 | 1 | 1 | 0.5 | 1 | 7 | Good |
| 73 | Qiu et al, 2020 | 1 | 1 | 0.5 | 1 | 0.5 | 0.5 | 0 | 0.5 | 0.5 | 1 | 6.5 | Fair |
| 74 | Shekerdemian et al, 2020 | 1 | 1 | 0.5 | 1 | 0.5 | 0 | 1 | 1 | 0.5 | 1 | 7.5 | Good |
| 75 | Zachariah et al, 2020 | 1 | 0.5 | 0 | 0.5 | 0.5 | 1 | 0 | 0 | 0.5 | 1 | 5 | Fair |
| 76 | Xiong et al, 2020 | 1 | 1 | 1 | 0 | 0.5 | 1 | 0 | 1 | 0 | 0.5 | 6 | Fair |
| 77 | Soltani et al, 2020 | 1 | 1 | 0.5 | 1 | 1 | 0.5 | 1 | 1 | 0.5 | 1 | 8.5 | Good |
| 78 | Somekh et al, 2020 | 1 | 1 | 0.5 | 0 | 1 | 0 | 0 | 0 | 0.5 | 0.5 | 4.5 | Poor |
| 79 | Zhao et al, 2020 | 1 | 1 | 0.5 | 0.5 | 0 | 0.5 | 1 | 1 | 0.5 | 1 | 7 | Good |
| 80 | Gao et al, 2020 | 1 | 1 | 1 | 1 | 1 | 1 | 0 | 1 | 1 | 0.5 | 8.5 | Good |
| 81 | Ma et al, 2020 | 1 | 1 | 0.5 | 0 | 1 | 1 | 1 | 1 | 0.5 | 1 | 8 | Good |
| 82 | Ma et al, 2020 | 1 | 1 | 0 | 0.5 | 0.5 | 0.5 | 1 | 1 | 0.5 | 1 | 7 | Good |
| 83 | Asfahan et al, 2020 | 1 | 0.5 | 1 | 0 | 0 | 0 | 0 | 1 | 1 | 1 | 5.5 | Fair |
| 84 | Zhang et al, 2020 | 1 | 1 | 0 | 0.5 | 0.5 | 0 | 0.5 | 1 | 0.5 | 1 | 6 | Fair |
| 85 | Zhao et al, 2020 | 1 | 1 | 0 | 0 | 0.5 | 0.5 | 0 | 1 | 0.5 | 0.5 | 5 | Fair |
| 86 | Shen et al, 2020 | 1 | 1 | 1 | 0 | 1 | 1 | 0 | 0 | 0.5 | 1 | 6.5 | Fair |
| 87 | Talarico et al, 2020 | 1 | 1 | 0.5 | 0 | 1 | 1 | 0 | 0 | 0.5 | 0.5 | 5.5 | Fair |
| 88 | DeBiasi et al, 2020 | 1 | 1 | 1 | 0 | 0 | 1 | 0 | 1 | 1 | 1 | 7 | Good |
| 89 | García-Salido 2020 | 1 | 1 | 1 | 0.5 | 1 | 1 | 1 | 1 | 1 | 1 | 9.5 | Good |
| 90 | Zhang et al, 2020 | 1 | 1 | 1 | 1 | 1 | 1 | 1 | 1 | 1 | 1 | 10 | Good |
| 91 | Liu et al, 2020 | 1 | 1 | 1 | 0.5 | 1 | 1 | 1 | 1 | 0.5 | 1 | 9 | Good |
| 92 | Lu et al, 2020 | 1 | 1 | 0.5 | 1 | 0.5 | 0 | 0 | 0 | 1 | 1 | 6 | Fair |
| 93 | Korkmaz et al, 2020 | 1 | 1 | 0.5 | 0.5 | 1 | 0 | 0.5 | 1 | 0.5 | 1 | 7 | Good |
| 94 | Han et al, 2020 | 0.5 | 1 | 0 | 1 | 1 | 1 | 0 | 0 | 1 | 1 | 6.5 | Fair |
| 95 | Zhang et al, 2020 | 1 | 1 | 1 | 0.5 | 0.5 | 1 | 1 | 1 | 0 | 1 | 8 | Good |
| 96 | Bai et al, 2020 | 1 | 1 | 0.5 | 1 | 1 | 0.5 | 1 | 1 | 0.5 | 1 | 8.5 | Good |
| 97 | Qualha et al, 2020 | 1 | 1 | 0 | 0 | 0.5 | 0.5 | 1 | 1 | 1 |  | 6 | Good |
| 98 | Peng et al, 2020 | 1 | 1 | 0 | 0 | 0.5 | 0.5 | 0.5 | 1 | 0.5 | 1 | 6 | Fair |
| 99 | Posfay-Barbe et al, 2020 | 1 | 1 | 0 | 0.5 | 1 | 1 | 1 | 0 | 0.5 | 1 | 7 | Good |

***Supplementary Table S4: Recruitment criteria* *for study participants in studies detailing asymptomatic infection***

| Author | Title | Recruitment criteria | Action |
| --- | --- | --- | --- |
| Mithal | SARS-CoV-2 Infection in Infants Less than 90 Days Old | Symptomatic | Exclude |
| Dodi | SARS-CoV-2 infection in children in Parma | Symptomatic or asymptomatic contact of confirmed case | Include |
| Sun | SARS-CoV-2 infection in infants under 1 year of age in Wuhan City, China | Tested on hospital admission | Exclude |
| Russell | Estimating the infection and case fatality ratio for coronavirus disease (COVID-19) using age-adjusted data from the outbreak on the Diamond Princess cruise ship, February 2020 | Whole population tested | Include |
| Hua | Epidemiological features and viral shedding in children with SARS‐CoV‐2 infection | Symptomatic or asymptomatic contact of confirmed case | Include |
| Du | Clinical characteristics of 182 pediatric COVID-19 patients with different severities and allergic status | Symptomatic or asymptomatic contact of confirmed case | Include |
| Zhao | Characteristics of Children With Reactivation of SARS-CoV-2 Infection After Hospital Discharge | Tested in hospital | Exclude |
| Song | Clinical features of pediatric patients with coronavirus disease (COVID-19) | Symptomatic or asymptomatic contact of confirmed case | Include |
| Peng | Coronavirus disease 2019 in children: Characteristics, antimicrobial treatment, and outcomes | Tested in hospital | Exclude |
| Lu | SARS-CoV-2 Infection in Children | Symptomatic or asymptomatic contact of confirmed case | Include |
| Lu | Symptomatic Infection is Associated with Prolonged Duration of Viral Shedding in Mild Coronavirus Disease 2019: A Retrospective Study of 110 Children in Wuhan | Symptomatic or asymptomatic contact of confirmed case | Include |
| Xu | Characteristics of pediatric SARS-CoV-2 infection and potential evidence for persistent fecal viral shedding | Symptomatic or asymptomatic contact of confirmed case | Include |
| Lu | Clinical characteristics and radiological features of children infected with the 2019 novel coronavirus | Symptomatic or asymptomatic contact of confirmed case | Include |
| Han | Viral RNA Load in Mildly Symptomatic and Asymptomatic Children with COVID-19, Seoul, South Korea | Tested in hospital | Exclude |
| Hildenwall | Paediatric COVID‐19 admissions in a region with open schools during the two first months of the pandemic | Tested in hospital | Exclude |
| Dong | Epidemiology of COVID-19 Among Children in China | Symptomatic or asymptomatic contact of confirmed case | Include |
| Parri | Children with Covid-19 in Pediatric Emergency Departments in Italy. | Tested in hospital | Exclude |
| Bai | Clinical Analysis of 25 Novel Coronavirus Infections in Children | Symptomatic or asymptomatic contact of confirmed case | Include |
| Zhang | Clinical Features of 33 Cases in Children Infected With SARS-CoV-2 in Anhui Province, China-A Multi-Center Retrospective Cohort Study | Symptomatic | Exclude |
| Xiong | A Comparison Between Chinese Children Infected with COVID-19 and with SARS | Symptomatic or asymptomatic contact of confirmed case | Include |
| Valente | Ocular manifestations and viral shedding in tears of pediatric patients with coronavirus disease 2019: a preliminary report | Symptomatic | Exclude |
| Parri | Characteristic of COVID-19 infection in pediatric patients: early findings from two Italian Pediatric Research Networks | Unclear | Exclude |
| Melgosa | SARS-CoV-2 infection in Spanish children with chronic kidney pathologies | Symptomatic or asymptomatic contact of confirmed case | Include |
| Ma | Characteristics of asymptomatic patients with SARS-CoV-2 infection in Jinan, China | Unclear | Exclude |
| Korkmaz | The Epidemiological and Clinical Characteristics of 81 Children with COVID-19 in a Pandemic Hospital in Turkey: an Observational Cohort | Symptomatic or asymptomatic contact of confirmed case | Include |
| De Loris | Dynamic Viral Severe Acute Respiratory Syndrome Coronavirus 2 RNA Shedding in Children: Preliminary Data and Clinical Consideration from a Italian Regional Center | Unclear | Exclude |
| Armann | Hospital Admission in Children and Adolescents With COVID-19 | Unclear | Exclude |
| Ma | A single-center, retrospective study of COVID-19 features in children: a descriptive investigation | Symptomatic or asymptomatic contact of confirmed case | Include |

## Section S3: Supplementary figures

Figure S1 shows the results of a metanalysis based on all studies contained in table S3; figure 3 in the main text shows only the results from “included” studies in table S3. The pooled estimate of asymptomatic children was 18.0% (95% CI: 13.0 - 22.1%) with a $\tau^{2}$ (between-trial-variance) of 0.009 (95%CI:0.004 - 0.022).


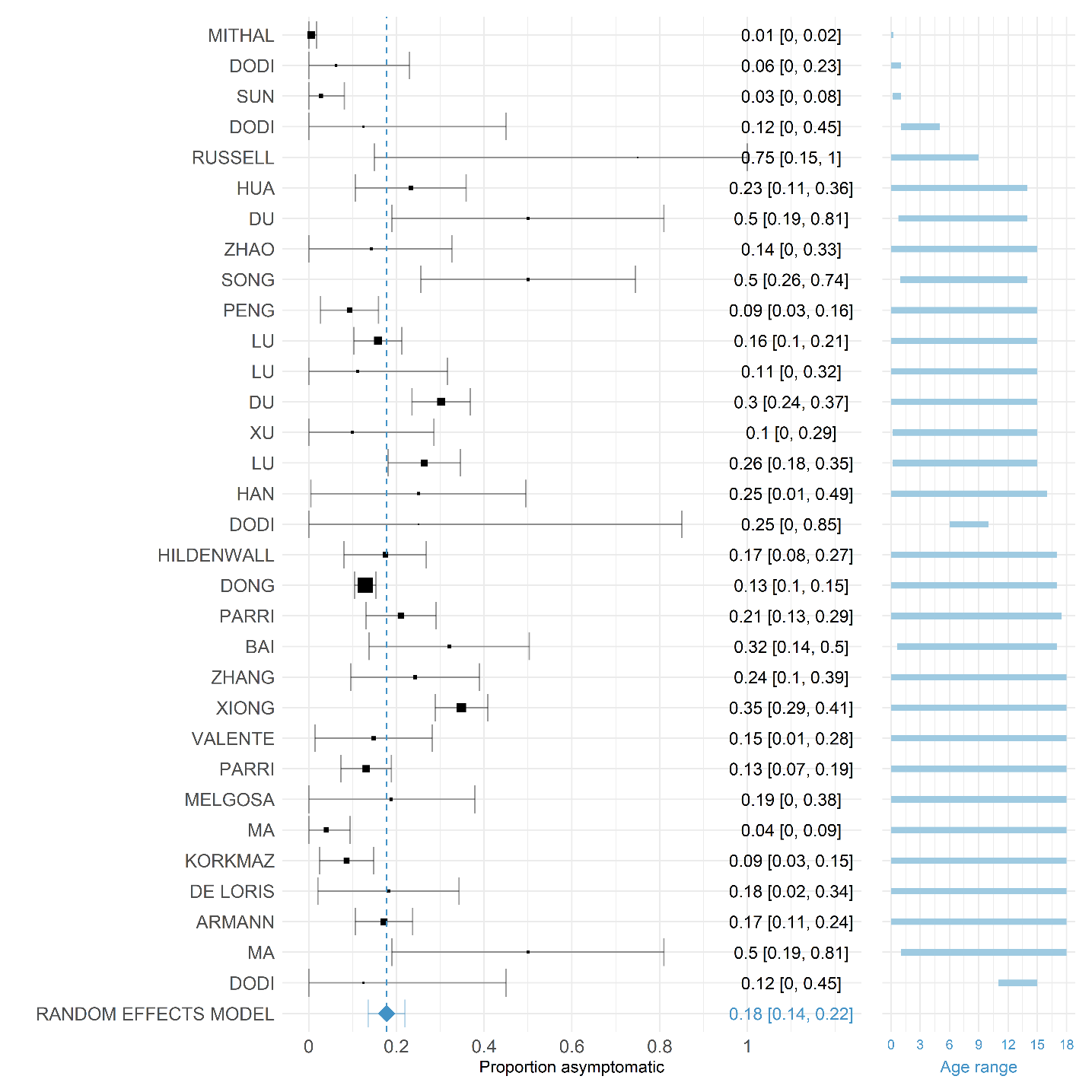


**Figure S1: Proportion of SARS-CoV-2 positive children who are defined as asymptomatic at the time of the study in each published study. The random effects model result is given at the bottom indicated by a blue diamond. The squares are proportional in size to the number of COVID-19 positive individuals in the study. All studies were conducted in 2020. The labels on the left provide first author, the labels on the right give point estimate and confidence interval of the asymptomatic proportion estimated. Studies are ordered by the mean of the age range with age range given in blue on the right.**


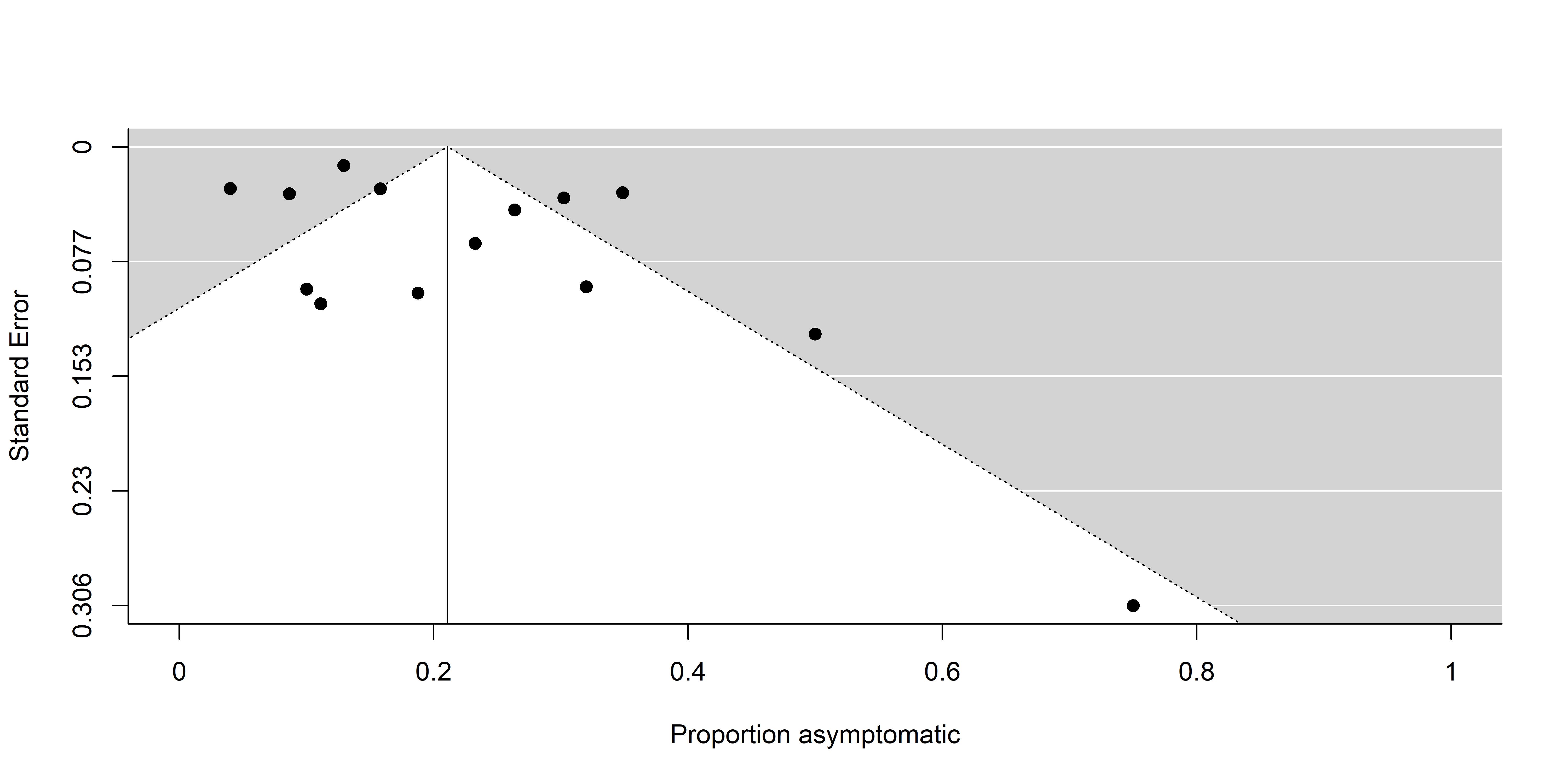
**Figure S2: Funnel plot associated with figure 3, the meta analysis of the proportion of asymptomatic COVID-19 positive children.**


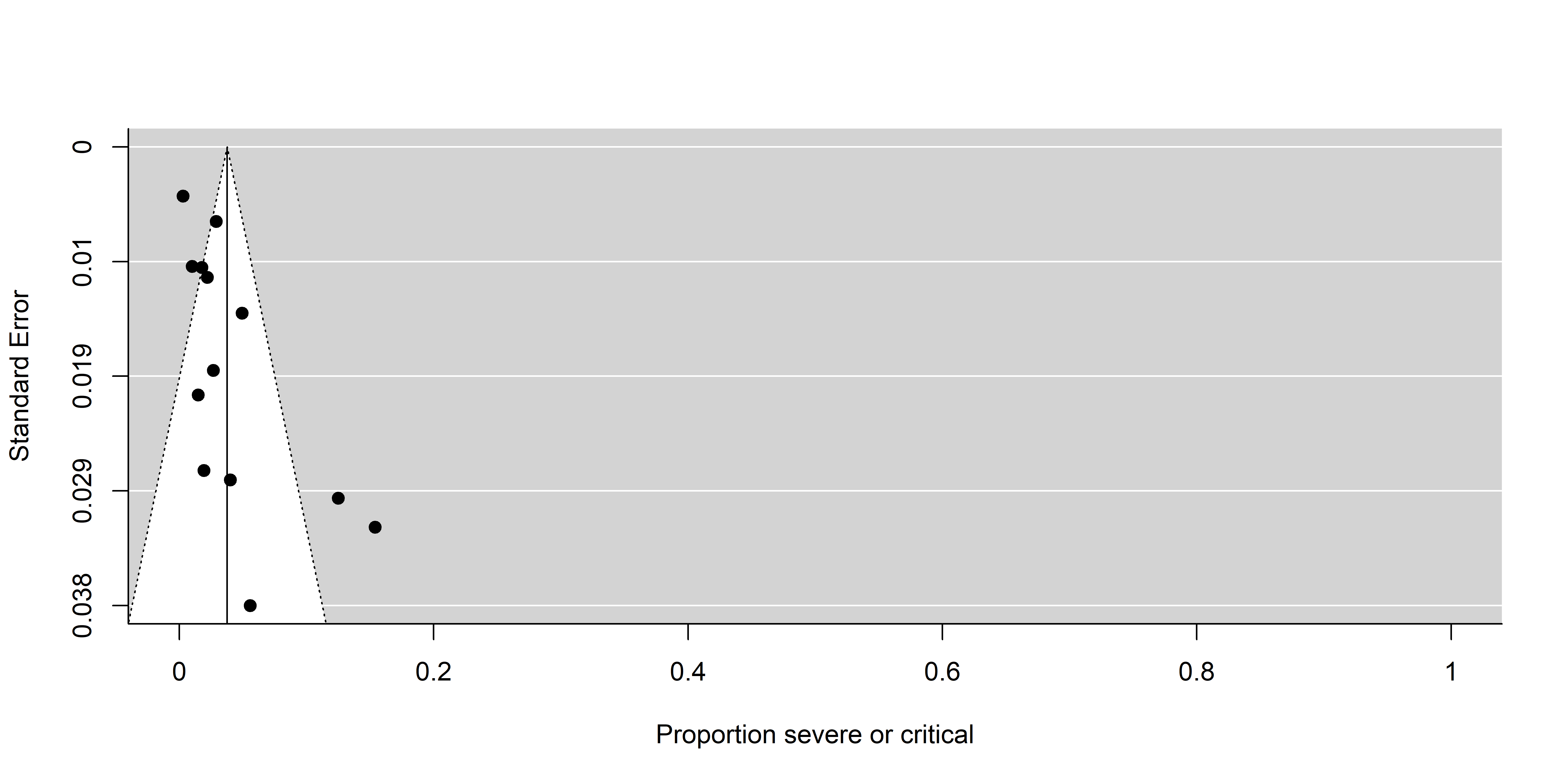


**Figure S3: Funnel plot associated with figure 4, the meta analysis of the proportion of COVID-19 positive children who are classified as severe or critical**

## Supplementary References

1. Armann JP, Diffloth N, Simon A, Doenhardt M, Hufnagel M, Trotter A, et al. Hospital Admission in Children and Adolescents With COVID-19. Dtsch Arztebl Int. 2020;117 21 PG-373–374:373–4. doi:10.3238/arztebl.2020.0373.

2. Asfahan S, Deokar K, Dutt N, Niwas R, Jain P, Agarwal M. Extrapolation of mortality in COVID-19: Exploring the role of age, sex, co-morbidities and health-care related occupation. Monaldi Arch Chest Dis. 2020;90 2 PG-. doi:10.4081/monaldi.2020.1325.

3. Bai K, Liu W, Liu C, Fu Y, Hu J, Qin Y, et al. Clinical Analysis of 25 Novel Coronavirus Infections in Children. Pediatr Infect Dis J. 2020; PG-. doi:10.1097/inf.0000000000002740.

4. Belhadjer Z, Méot M, Bajolle F, Khraiche D, Legendre A, Abakka S, et al. Acute heart failure in multisystem inflammatory syndrome in children (MIS-C) in the context of global SARS-CoV-2 pandemic. Circulation. 2020; PG-. doi:10.1161/circulationaha.120.048360.

5. Belot A, Antona D, Renolleau S, Javouhey E, Hentgen V, Angoulvant F, et al. SARS-CoV-2-related paediatric inflammatory multisystem syndrome, an epidemiological study, France, 1 March to 17 May 2020. Euro Surveill. 2020;25 22 PG-. doi:10.2807/1560-7917.Es.2020.25.22.2001010.

6. Bi Q, Wu Y, Mei S, Ye C, Zou X, Zhang Z, et al. Epidemiology and transmission of COVID-19 in 391 cases and 1286 of their close contacts in Shenzhen, China: a retrospective cohort study. Lancet Infect Dis. 2020; PG-. doi:10.1016/s1473-3099(20)30287-5.

7. Brambilla I, Castagnoli R, Caimmi S, Ciprandi G, Luigi Marseglia G. COVID-19 in the Pediatric Population Admitted to a Tertiary Referral Hospital in Northern Italy: Preliminary Clinical Data. Pediatr Infect Dis J. 2020;39 7 PG-e160:e160. doi:10.1097/inf.0000000000002730.

8. Buonsenso D, Costa S, Sanguinetti M, Cattani P, Posteraro B, Marchetti S, et al. Neonatal Late Onset Infection with Severe Acute Respiratory Syndrome Coronavirus 2. Am J Perinatol. 2020; PG-. doi:10.1055/s-0040-1710541.

9. Cai J, Xu J, Lin D, Yang Z, Xu L, Qu Z, et al. A Case Series of children with 2019 novel coronavirus infection: clinical and epidemiological features. Clin Infect Dis. 2020; PG-. doi:10.1093/cid/ciaa198.

10. Chen J, Zhang ZZ, Chen YK, Long QX, Tian WG, Deng HJ, et al. The clinical and immunological features of pediatric COVID-19 patients in China. Genes Dis. 2020; PG-. doi:10.1016/j.gendis.2020.03.008.

11. Chen Z, Tong L, Zhou Y, Hua C, Wang W, Fu J, et al. Childhood COVID-19: a multi-center retrospective study. Clin Microbiol Infect. 2020; PG-. doi:10.1016/j.cmi.2020.06.015.

12. Danis K, Epaulard O, Bénet T, Gaymard A, Campoy S, Bothelo-Nevers E, et al. Cluster of coronavirus disease 2019 (Covid-19) in the French Alps, 2020. Clin Infect Dis. 2020; PG-. doi:10.1093/cid/ciaa424.

13. DeBiasi RL, Song X, Delaney M, Bell M, Smith K, Pershad J, et al. Severe COVID-19 in Children and Young Adults in the Washington, DC Metropolitan Region. J Pediatr. 2020; PG-. doi:10.1016/j.jpeds.2020.05.007.

14. de Ceano-Vivas M, Martín-Espín I, Del Rosal T, Bueno-Barriocanal M, Plata-Gallardo M, Ruiz-Domínguez JA, et al. SARS-CoV-2 infection in ambulatory and hospitalised Spanish children. Arch Dis Child. 2020; PG-. doi:10.1136/archdischild-2020-319366.

15. De Ioris MA, Scarselli A, Ciofi Degli Atti ML, Ravà L, Smarrazzo A, Concato C, et al. Dynamic viral SARS-CoV-2 RNA shedding in in children: preliminary data and clinical consideration of Italian regional center. J Pediatr Infect Dis Soc. 2020; PG-. doi:10.1093/jpids/piaa065.

16. de Lusignan S, Dorward J, Correa A, Jones N, Akinyemi O, Amirthalingam G, et al. Risk factors for SARS-CoV-2 among patients in the Oxford Royal College of General Practitioners Research and Surveillance Centre primary care network: a cross-sectional study. Lancet Infect Dis. 2020; PG-. doi:10.1016/s1473-3099(20)30371-6.

17. Dimeglio C, Mansuy JM, Charpentier S, Claudet I, Izopet J. Children are protected against SARS-CoV-2 infection. J Clin Virol. 2020;128 PG-104451:104451. doi:10.1016/j.jcv.2020.104451.

18. Docherty AB, Harrison EM, Green CA, Hardwick HE, Pius R, Norman L, et al. Features of 20 133 UK patients in hospital with covid-19 using the ISARIC WHO Clinical Characterisation Protocol: prospective observational cohort study. Bmj. 2020;369 PG-m1985:m1985. doi:10.1136/bmj.m1985.

19. Dodi I, Castellone E, Pappalardo M, Rubini M, Veronese P, Ruberto C, et al. SARS-CoV-2 infection in children in Parma. Acta Biomed. 2020;91 2 PG-214–215:214–5. doi:10.23750/abm.v91i2.9563.

20. Dong Y, Dong Y, Mo X, Hu Y, Qi X, Jiang F, et al. Epidemiology of COVID-19 among children in China. Pediatrics. 2020; PG-. doi:10.1542/peds.2020-0702.

21. Dong X, Cao YY, Lu XX, Zhang JJ, Du H, Yan YQ, et al. Eleven faces of coronavirus disease 2019. Allergy. 2020; PG-. doi:10.1111/all.14289.

22. Du W, Yu J, Wang H, Zhang X, Zhang S, Li Q, et al. Clinical characteristics of COVID-19 in children compared with adults in Shandong Province, China. Infection. 2020; PG-1-8:1–8. doi:10.1007/s15010-020-01427-2.

23. Du W, Yu J, Liu X, Chen H, Lin L, Li Q. Persistence of SARS-CoV-2 virus RNA in feces: A case series of children. J Infect Public Heal. 2020;13 7 PG-926–31:926–31. doi:10.1016/j.jiph.2020.05.025.

24. Du H, Dong X, Zhang J jin, Cao Y yuan, Akdis M, Huang P qi, et al. Clinical characteristics of 182 pediatric COVID-19 patients with different severities and allergic status. Allergy. 2020; PG-. doi:10.1111/all.14452.

25. Dufort EM, Koumans EH, Chow EJ, Rosenthal EM, Muse A, Rowlands J, et al. Multisystem Inflammatory Syndrome in Children in New York State. N Engl J Med. 2020; PG-. doi:10.1056/NEJMoa2021756.

26. Foster CE, Moulton EA, Munoz FM, Hulten KG, Versalovic J, Dunn J, et al. Coronavirus Disease 2019 in Children Cared for at Texas Children’s Hospital: Initial Clinical Characteristics and Outcomes. J Pediatr Infect Dis Soc. 2020; PG-. doi:10.1093/jpids/piaa072.

27. Gao Q, Hu Y, Dai Z, Xiao F, Wang J, Wu J. The epidemiological characteristics of 2019 novel coronavirus diseases (COVID-19) in Jingmen, Hubei, China. Med. 2020;99 23 PG-e20605:e20605. doi:10.1097/md.0000000000020605.

28. Garazzino S, Montagnani C, Donà D, Meini A, Felici E, Vergine G, et al. Multicentre Italian study of SARS-CoV-2 infection in children and adolescents, preliminary data as at 10 April 2020. Euro Surveill. 2020;25 18 PG-. doi:10.2807/1560-7917.Es.2020.25.18.2000600.

29. García-Salido A, Leoz-Gordillo I, Martínez de Azagra-Garde A, Nieto-Moro M, Iglesias-Bouzas MI, García-Teresa MÁ, et al. Children in Critical Care Due to Severe Acute Respiratory Syndrome Coronavirus 2 Infection: Experience in a Spanish Hospital. Pediatr Crit Care Med. 2020; PG-. doi:10.1097/pcc.0000000000002475.

30. González Cortés R, García-Salido A, Roca Pascual D, Slöcker Barrio M, de Carlos Vicente JC. A multicenter national survey of children with SARS-CoV-2 infection admitted to Spanish Pediatric Intensive Care Units. Intensive Care Med. 2020; PG-1-3:1–3. doi:10.1007/s00134-020-06146-8.

31. Götzinger F, Santiago-García B, Noguera-Julián A, Lanaspa M, Lancella L, Calò Carducci FI, et al. COVID-19 in children and adolescents in Europe: a multinational, multicentre cohort study. Lancet Child Adolesc Heal. 2020; PG-. doi:10.1016/s2352-4642(20)30177-2.

32. Guan WJ, Ni ZY, Hu Y, Liang WH, Ou CQ, He JX, et al. Clinical Characteristics of Coronavirus Disease 2019 in China. N Engl J Med. 2020;382 18 PG-1708–1720:1708–20. doi:10.1056/NEJMoa2002032.

33. Gudbjartsson DF, Helgason A, Jonsson H, Magnusson OT, Melsted P, Norddahl GL, et al. Spread of SARS-CoV-2 in the Icelandic Population. N Engl J Med. 2020; PG-. doi:10.1056/NEJMoa2006100.

34. Gujski M, Raciborski F, Jankowski M, Nowicka PM, Rakocy K, Pinkas J. Epidemiological Analysis of the First 1389 Cases of COVID-19 in Poland: A Preliminary Report. Med Sci Monit. 2020;26 PG-e924702:e924702. doi:10.12659/msm.924702.

35. Han YN, Feng ZW, Sun LN, Ren XX, Wang H, Xue YM, et al. A comparative-descriptive analysis of clinical characteristics in 2019-coronavirus-infected children and adults. J Med Virol. 2020; PG-. doi:10.1002/jmv.25835.

36. Han MS, Seong MW, Kim N, Shin S, Cho SI, Park H, et al. Viral RNA Load in Mildly Symptomatic and Asymptomatic Children with COVID-19, Seoul. Emerg Infect Dis. 2020;26 10 PG-. doi:10.3201/eid2610.202449.

37. Harman K, Verma A, Cook J, Radia T, Zuckerman M, Deep A, et al. Ethnicity and COVID-19 in children with comorbidities. Lancet Child Adolesc Heal. 2020;4 7 PG-24–25:e24–5. doi:10.1016/s2352-4642(20)30167-x.

38. He G, Sun W, Fang P, Huang J, Gamber M, Cai J, et al. The clinical feature of silent infections of novel coronavirus infection (COVID-19) in Wenzhou. J Med Virol. 2020; PG-. doi:10.1002/jmv.25861.

39. Heavey L, Casey G, Kelly C, Kelly D, McDarby G. No evidence of secondary transmission of COVID-19 from children attending school in Ireland, 2020. Euro Surveill. 2020;25 21 PG-. doi:10.2807/1560-7917.Es.2020.25.21.2000903.

40. Hildenwall H, Luthander J, Rhedin S, Hertting O, Olsson-Åkefeldt S, Melén E, et al. Paediatric COVID-19 admissions in a region with open schools during the two first months of the pandemic. Acta Paediatr. 2020; PG-. doi:10.1111/apa.15432.

41. Hrusak O, Kalina T, Wolf J, Balduzzi A, Provenzi M, Rizzari C, et al. Flash survey on severe acute respiratory syndrome coronavirus-2 infections in paediatric patients on anticancer treatment. Eur J Cancer. 2020;132 PG-11-16:11–6. doi:10.1016/j.ejca.2020.03.021.

42. Hu Z, Song C, Xu C, Jin G, Chen Y, Xu X, et al. Clinical characteristics of 24 asymptomatic infections with COVID-19 screened among close contacts in Nanjing, China. Sci China Life Sci. 2020;63 5 PG-706–711:706–11. doi:10.1007/s11427-020-1661-4.

43. Hua CZ, Miao ZP, Zheng JS, Huang Q, Sun QF, Lu HP, et al. Epidemiological features and viral shedding in children with SARS-CoV-2 infection. J Med Virol. 2020; PG-. doi:10.1002/jmv.26180.

44. Ibrahim LF, Tosif S, McNab S, Hall S, Lee HJ, Lewena S, et al. SARS-CoV-2 testing and outcomes in the first 30 days after the first case of COVID-19 at an Australian children’s hospital. Emerg Med Australas. 2020; PG-. doi:10.1111/1742-6723.13550.

45. Ji T, Chen HL, Xu J, Wu LN, Li JJ, Chen K, et al. Lockdown contained the spread of 2019 novel coronavirus disease in Huangshi city, China: Early epidemiological findings. Clin Infect Dis. 2020; PG-. doi:10.1093/cid/ciaa390.

46. Report on the Epidemiological Features of Coronavirus Disease 2019 (COVID-19) Outbreak in the Republic of Korea from January 19 to March 2, 2020. J Korean Med Sci. 2020;35 10 PG-e112:e112. doi:10.3346/jkms.2020.35.e112.

47. Korkmaz MF, Türe E, Dorum BA, Kılıç ZB. The Epidemiological and Clinical Characteristics of 81 Children with COVID-19 in a Pandemic Hospital in Turkey: an Observational Cohort Study. J Korean Med Sci. 2020;35 25 PG-e236:e236. doi:10.3346/jkms.2020.35.e236.

48. L’Huillier AG, Torriani G, Pigny F, Kaiser L, Eckerle I. Culture-Competent SARS-CoV-2 in Nasopharynx of Symptomatic Neonates, Children, and Adolescents. Emerg Infect Dis. 2020;26 10 PG-. doi:10.3201/eid2610.202403.

49. Lavezzo E, Franchin E, Ciavarella C, Cuomo-Dannenburg G, Barzon L, Del Vecchio C, et al. Suppression of a SARS-CoV-2 outbreak in the Italian municipality of Vo’. Nature. 2020; PG-. doi:10.1038/s41586-020-2488-1.

50. Li W, Zhang B, Lu J, Liu S, Chang Z, Cao P, et al. The characteristics of household transmission of COVID-19. Clin Infect Dis. 2020; PG-. doi:10.1093/cid/ciaa450.

51. Li H, Chen K, Liu M, Xu H, Xu Q. The profile of peripheral blood lymphocyte subsets and serum cytokines in children with 2019 novel coronavirus pneumonia. J Infect. 2020; PG-. doi:10.1016/j.jinf.2020.04.001.

52. Li Y, Wang H, Wang F, Du H, Liu X, Chen P, et al. Comparison of Hospitalized Patients with pneumonia caused by COVID-19 and influenza A in children under 5 years. Int J Infect Dis. 2020; PG-. doi:10.1016/j.ijid.2020.06.026.

53. Liu W, Zhang Q, Chen J, Xiang R, Song H, Shu S, et al. Detection of Covid-19 in Children in Early January 2020 in Wuhan, China. N Engl J Med. 2020;382 14 PG-1370–1371:1370–1. doi:10.1056/NEJMc2003717.

54. Liu H, Liu F, Li J, Zhang T, Wang D, Lan W. Clinical and CT imaging features of the COVID-19 pneumonia: Focus on pregnant women and children. J Infect. 2020;80 5 PG-7–13:e7–13. doi:10.1016/j.jinf.2020.03.007.

55. Liu J, Liao X, Qian S, Yuan J, Wang F, Liu Y, et al. Community Transmission of Severe Acute Respiratory Syndrome Coronavirus 2, Shenzhen, China, 2020. Emerg Infect Dis. 2020;26 6 PG-1320–1323:1320–3. doi:10.3201/eid2606.200239.

56. Liu T, Liang W, Zhong H, He J, Chen Z, He G, et al. Risk factors associated with COVID-19 infection: a retrospective cohort study based on contacts tracing. Emerg Microbes Infect. 2020; PG-1-31:1–31. doi:10.1080/22221751.2020.1787799.

57. Lu X, Zhang L, Du H, Zhang J, Li YYY, Qu J, et al. SARS-CoV-2 infection in children. N Engl J Med. 2020;382 17 PG-1663–1665:1663–5. doi:10.1056/NEJMc2005073.

58. Lu Y, Wen H, Rong D, Zhou Z, Liu H. Clinical characteristics and radiological features of children infected with the 2019 novel coronavirus. Clin Radiol. 2020;75 7 PG-520–525:520–5. doi:10.1016/j.crad.2020.04.010.

59. Lu Y, Li Y, Deng W, Liu M, He Y, Huang L, et al. Symptomatic Infection is Associated with Prolonged Duration of Viral Shedding in Mild Coronavirus Disease 2019: A Retrospective Study of 110 Children in Wuhan. Pediatr Infect Dis J. 2020;39 7 PG-95–99:e95–9. doi:10.1097/inf.0000000000002729.

60. Ma Y, Xu QN, Wang FL, Ma XM, Wang XY, Zhang XG, et al. Characteristics of asymptomatic patients with SARS-CoV-2 infection in Jinan, China. Microbes Infect. 2020;22:212–7. doi:10.1016/j.micinf.2020.04.011.

61. Ma H, Hu J, Tian J, Zhou X, Li H, Laws MT, et al. A single-center, retrospective study of COVID-19 features in children: a descriptive investigation. BMC Med. 2020;18 1 PG-123:123. doi:10.1186/s12916-020-01596-9.

62. Mannheim J, Gretsch S, Layden JE, Fricchione MJ. Characteristics of Hospitalized Pediatric COVID-19 Cases - Chicago, Illinois, March - April 2020. J Pediatr Infect Dis Soc. 2020; PG-. doi:10.1093/jpids/piaa070.

63. Marlais M, Wlodkowski T, Vivarelli M, Pape L, Tönshoff B, Schaefer F, et al. The severity of COVID-19 in children on immunosuppressive medication. Lancet Child Adolesc Heal. 2020;4 7 PG-17–18:e17–8. doi:10.1016/s2352-4642(20)30145-0.

64. Martínez-Perez O, Vouga M, Cruz Melguizo S, Forcen Acebal L, Panchaud A, Muñoz-Chápuli M, et al. Association Between Mode of Delivery Among Pregnant Women With COVID-19 and Maternal and Neonatal Outcomes in Spain. Jama. 2020; PG-. doi:10.1001/jama.2020.10125.

65. Melgosa M, Madrid A, Alvárez O, Lumbreras J, Nieto F, Parada E, et al. SARS-CoV-2 infection in Spanish children with chronic kidney pathologies. Pediatr Nephrol. 2020;35 8 PG-1521–1524:1521–4. doi:10.1007/s00467-020-04597-1.

66. Mithal LB, Machut KZ, Muller WJ, Kociolek LK. SARS-CoV-2 Infection in Infants Less than 90 Days Old. J Pediatr. 2020; PG-.

67. Mizumoto K, Kagaya K, Zarebski A, Chowell G. Estimating the asymptomatic proportion of coronavirus disease 2019 (COVID-19) cases on board the Diamond Princess cruise ship, Yokohama, Japan, 2020. Euro Surveill. 2020;25 10 PG-. doi:10.2807/1560-7917.Es.2020.25.10.2000180.

68. Moratto D, Giacomelli M, Chiarini M, Savarè L, Saccani B, Motta M, et al. Immune response in children with COVID-19 is characterized by lower levels of T cell activation than infected adults. Eur J Immunol. 2020; PG-. doi:10.1002/eji.202048724.

69. Ng OT, Marimuthu K, Chia PY, Koh V, Chiew CJ, De Wang L, et al. SARS-CoV-2 Infection among Travelers Returning from Wuhan, China. N Engl J Med. 2020;382 15 PG-1476–1478:1476–8. doi:10.1056/NEJMc2003100.

70. Otto WR, Geoghegan S, Posch LC, Bell LM, Coffin SE, Sammons JS, et al. The Epidemiology of SARS-CoV-2 in a Pediatric Healthcare Network in the United States. J Pediatr Infect Dis Soc. 2020; PG-. doi:10.1093/jpids/piaa074.

71. Oualha M, Bendavid M, Berteloot L, Corsia A, Lesage F, Vedrenne M, et al. Severe and fatal forms of COVID-19 in children. Arch Pediatr. 2020;27 5 PG-235–238:235–8. doi:10.1016/j.arcped.2020.05.010.

72. Pan A, Liu L, Wang C, Guo H, Hao X, Wang Q, et al. Association of Public Health Interventions With the Epidemiology of the COVID-19 Outbreak in Wuhan, China. Jama. 2020; PG-. doi:10.1001/jama.2020.6130.

73. Park YJ, Choe YJ, Park O, Park SY, Kim Y-M, Kim J, et al. Contact Tracing during Coronavirus Disease Outbreak, South Korea, 2020. Emerg Infect Dis J. 2020;26 10 PG-. doi:10.3201/eid2610.201315.

74. Parri N, Lenge M, Buonsenso D. Children with Covid-19 in Pediatric Emergency Departments in Italy. N Engl J Med. 2020; PG-. doi:10.1056/NEJMc2007617.

75. Parri N, Magistà AM, Marchetti F, Cantoni B, Arrighini A, Romanengo M, et al. Characteristic of COVID-19 infection in pediatric patients: early findings from two Italian Pediatric Research Networks. Eur J Pediatr. 2020; PG-1-9:1–9. doi:10.1007/s00431-020-03683-8.

76. Pathak EB, Salemi JL, Sobers N, Menard J, Hambleton IR. COVID-19 in Children in the United States: Intensive Care Admissions, Estimated Total Infected, and Projected Numbers of Severe Pediatric Cases in 2020. J Public Heal Manag Pr. 2020; PG-. doi:10.1097/phh.0000000000001190.

77. Peng H, Gao P, Xu Q, Liu M, Peng J, Wang Y, et al. Coronavirus disease 2019 in children: Characteristics, antimicrobial treatment, and outcomes. J Clin Virol. 2020;128 PG-104425:104425. doi:10.1016/j.jcv.2020.104425.

78. Posfay-Barbe KM, Wagner N, Gauthey M, Moussaoui D, Loevy N, Diana A, et al. COVID-19 in Children and the Dynamics of Infection in Families. Pediatrics. 2020; PG-. doi:10.1542/peds.2020-1576.

79. Pouletty M, Borocco C, Ouldali N, Caseris M, Basmaci R, Lachaume N, et al. Paediatric multisystem inflammatory syndrome temporally associated with SARS-CoV-2 mimicking Kawasaki disease (Kawa-COVID-19): a multicentre cohort. Ann Rheum Dis. 2020; PG-. doi:10.1136/annrheumdis-2020-217960.

80. Preßler J, Fill Malfertheiner S, Kabesch M, Buntrock-Döpke H, Häusler S, Ambrosch A, et al. Postnatal SARS-CoV-2 infection and immunological reaction: A prospective family cohort study. Pediatr Allergy Immunol. 2020; PG-. doi:10.1111/pai.13302.

81. Qiu H, Wu J, Hong L, Luo Y, Song Q, Chen D. Clinical and epidemiological features of 36 children with coronavirus disease 2019 (COVID-19) in Zhejiang, China: an observational cohort study. Lancet Infect Dis. 2020; PG-. doi:10.1016/s1473-3099(20)30198-5.

82. Qiu C, Cui C, Hautefort C, Haehner A, Zhao J, Yao Q, et al. Olfactory and Gustatory Dysfunction as an Early Identifier of COVID-19 in Adults and Children: An International Multicenter Study. Otolaryngol Head Neck Surg. 2020; PG-194599820934376:194599820934376. doi:10.1177/0194599820934376.

83. Ranabothu S, Onteddu S, Nalleballe K, Dandu V, Veerapaneni K, Veerapandiyan A. Spectrum of COVID-19 in Children. Acta Paediatr. 2020; PG-. doi:10.1111/apa.15412.

84. Rezaei N. COVID-19 affects healthy pediatricians more than pediatric patients. Infect Control Hosp Epidemiol. 2020; PG-1:1. doi:10.1017/ice.2020.139.

85. Rha B, Lively JY, Englund JA, Staat MA, Weinberg GA, Selvarangan R, et al. SARS-CoV-2 Infections in Children - Multi-Center Surveillance, United States, January-March 2020. J Pediatr Infect Dis Soc. 2020; PG-. doi:10.1093/jpids/piaa075.

86. Russell TW, Hellewell J, Jarvis CI, van Zandvoort K, Abbott S, Ratnayake R, et al. Estimating the infection and case fatality ratio for coronavirus disease (COVID-19) using age-adjusted data from the outbreak on the Diamond Princess cruise ship, February 2020. Euro Surveill. 2020;25 12 PG-. doi:10.2807/1560-7917.Es.2020.25.12.2000256.

87. Schwierzeck V, König JC, Kühn J, Mellmann A, Correa-Martínez CL, Omran H, et al. First reported nosocomial outbreak of severe acute respiratory syndrome coronavirus 2 (SARS-CoV-2) in a pediatric dialysis unit. Clin Infect Dis. 2020; PG-. doi:10.1093/cid/ciaa491.

88. Shekerdemian LS, Mahmood NR, Wolfe KK, Riggs BJ, Ross CE, McKiernan CA, et al. Characteristics and Outcomes of Children With Coronavirus Disease 2019 (COVID-19) Infection Admitted to US and Canadian Pediatric Intensive Care Units. JAMA Pediatr. 2020; PG-. doi:10.1001/jamapediatrics.2020.1948.

89. Shen N, Zhu Y, Wang X, Peng J, Liu W, Wang F, et al. Characteristics and diagnosis rate of 5630 subjects receiving SARS-CoV-2 nucleic acid tests from Wuhan, China. JCI Insight. 2020;5 10 PG-. doi:10.1172/jci.insight.137662.

90. Shi Y, Wang X, Liu G, Zhu Q, Wang J, Yu H, et al. A quickly, effectively screening process of novel corona virus disease 2019 (COVID-19) in children in Shanghai, China. Ann Transl Med. 2020;8 5 PG-241:241. doi:10.21037/atm.2020.03.22.

91. Soltani J, Sedighi I, Shalchi Z, Sami G, Moradveisi B, Nahidi S. Pediatric coronavirus disease 2019 (COVID-19): An insight from west of Iran. North Clin Istanb. 2020;7 3 PG-284–291:284–91. doi:10.14744/nci.2020.90277.

92. Somekh E, Gleyzer A, Heller E, Lopian M, Kashani-Ligumski L, Czeiger S, et al. The Role of Children in the Dynamics of Intra Family Coronavirus 2019 Spread in Densely Populated Area. Pediatr Infect Dis J. 2020; PG-. doi:10.1097/inf.0000000000002783.

93. Son MBF. Pediatric inflammatory syndrome temporally related to covid-19. Bmj. 2020;369 PG-m2123:m2123. doi:10.1136/bmj.m2123.

94. Song W, Li J, Zou N, Guan W, Pan J, Xu W. Clinical features of pediatric patients with coronavirus disease (COVID-19). J Clin Virol. 2020;127 PG-104377:104377. doi:10.1016/j.jcv.2020.104377.

95. Song R, Han B, Song M, Wang L, Conlon CP, Dong T, et al. Clinical and epidemiological features of COVID-19 family clusters in Beijing, China. J Infect. 2020; PG-. doi:10.1016/j.jinf.2020.04.018.

96. Spiteri G, Fielding J, Diercke M, Campese C, Enouf V, Gaymard A, et al. First cases of coronavirus disease 2019 (COVID-19) in the WHO European Region, 24 January to 21 February 2020. Euro Surveill. 2020;25 9 PG-. doi:10.2807/1560-7917.Es.2020.25.9.2000178.

97. Stringhini S, Wisniak A, Piumatti G, Azman AS, Lauer SA, Baysson H, et al. Seroprevalence of anti-SARS-CoV-2 IgG antibodies in Geneva, Switzerland (SEROCoV-POP): a population-based study. Lancet. 2020.

98. Sun K, Chen J, Viboud C. Early epidemiological analysis of the coronavirus disease 2019 outbreak based on crowdsourced data: a population-level observational study. Lancet Digit Heal. 2020;2 4 PG-201–208:e201–8. doi:10.1016/s2589-7500(20)30026-1.

99. Sun D, Chen X, Li H, Lu XX, Xiao H, Zhang FR, et al. SARS-CoV-2 infection in infants under 1 year of age in Wuhan City, China. World J Pediatr. 2020;16 3 PG-260–266:260–6. doi:10.1007/s12519-020-00368-y.

100. Talarico V, Nicoletti A, Sabetta L, Minchella P, Raiola G. Preliminary epidemiological analysis on children and adolescents with novel coronavirus disease (2019-nCoV) in a central area of Calabria region. Acta Biomed. 2020;91 2 PG-232–233:232–3. doi:10.23750/abm.v91i2.9550.

101. Tan YP, Tan BY, Pan J, Wu J, Zeng SZ, Wei HY. Epidemiologic and clinical characteristics of 10 children with coronavirus disease 2019 in Changsha, China. J Clin Virol. 2020;127 PG-104353:104353. doi:10.1016/j.jcv.2020.104353.

102. Tian S, Hu N, Lou J, Chen K, Kang X, Xiang Z, et al. Characteristics of COVID-19 infection in Beijing. J Infect. 2020;80 4 PG-401–406:401–6. doi:10.1016/j.jinf.2020.02.018.

103. Tong ZD, Tang A, Li KF, Li P, Wang HL, Yi JP, et al. Potential Presymptomatic Transmission of SARS-CoV-2, Zhejiang Province, China, 2020. Emerg Infect Dis. 2020;26 5 PG-1052–1054:1052–4. doi:10.3201/eid2605.200198.

104. Turner D, Huang Y, Martín-de-Carpi J, Aloi M, Focht G, Kang B, et al. COVID-19 and Paediatric Inflammatory Bowel Diseases: Global Experience and Provisional Guidance (March 2020) from the Paediatric IBD Porto group of ESPGHAN. J Pediatr Gastroenterol Nutr. 2020; PG-. doi:10.1097/mpg.0000000000002729.

105. Valente P, Iarossi G, Federici M, Petroni S, Palma P, Cotugno N, et al. Ocular manifestations and viral shedding in tears of pediatric patients with coronavirus disease 2019: a preliminary report. J aapos. 2020; PG-. doi:10.1016/j.jaapos.2020.05.002.

106. Verdoni L, Mazza A, Gervasoni A, Martelli L, Ruggeri M, Ciuffreda M, et al. An outbreak of severe Kawasaki-like disease at the Italian epicentre of the SARS-CoV-2 epidemic: an observational cohort study. Lancet. 2020;395 10239 PG-1771–1778:1771–8. doi:10.1016/s0140-6736(20)31103-x.

107. Wu JT, Leung K, Bushman M, Kishore N, Niehus R, de Salazar PM, et al. Estimating clinical severity of COVID-19 from the transmission dynamics in Wuhan, China. Nat Med. 2020;26 4 PG-506–510:506–10. doi:10.1038/s41591-020-0822-7.

108. Wu Q, Xing Y, Shi L, Li W, Gao Y, Pan S, et al. Coinfection and Other Clinical Characteristics of COVID-19 in Children. Pediatrics. 2020;146 1 PG-. doi:10.1542/peds.2020-0961.

109. Wu H, Zhu H, Yuan C, Yao C, Luo W, Shen X, et al. Clinical and Immune Features of Hospitalized Pediatric Patients With Coronavirus Disease 2019 (COVID-19) in Wuhan, China. JAMA Netw Open. 2020;3 6 PG-e2010895:e2010895. doi:10.1001/jamanetworkopen.2020.10895.

110. Xia W, Shao J, Guo Y, Peng X, Li Z, Hu D. Clinical and CT features in pediatric patients with COVID-19 infection: Different points from adults. Pediatr Pulmonol. 2020;55 5 PG-1169–1174:1169–74. doi:10.1002/ppul.24718.

111. Xiong XL, Wong KK, Chi SQ, Zhou AF, Tang JQ, Zhou LS, et al. Comparative study of the clinical characteristics and epidemiological trend of 244 COVID-19 infected children with or without GI symptoms. Gut. 2020; PG-. doi:10.1136/gutjnl-2020-321486.

112. Xiong X, Chua GT, Chi S, Wah Kwan MY, Sang Wong WH, Zhou A, et al. A Comparison Between Chinese Children Infected with COVID-19 and with SARS. J Pediatr. 2020; PG-. doi:10.1016/j.jpeds.2020.06.041.

113. Xu Y, Li X, Zhu B, Liang H, Fang C, Gong Y, et al. Characteristics of pediatric SARS-CoV-2 infection and potential evidence for persistent fecal viral shedding. Nat Med. 2020;26 4 PG-502–505:502–5. doi:10.1038/s41591-020-0817-4.

114. Xu XW, Wu XX, Jiang XG, Xu KJ, Ying LJ, Ma CL, et al. Clinical findings in a group of patients infected with the 2019 novel coronavirus (SARS-Cov-2) outside of Wuhan, China: retrospective case series. Bmj. 2020;368 PG-m606:m606. doi:10.1136/bmj.m606.

115. Xu B, Gutierrez B, Mekaru S, Sewalk K, Goodwin L, Loskill A, et al. Epidemiological data from the COVID-19 outbreak, real-time case information. Sci Data. 2020;7 1 PG-106:106. doi:10.1038/s41597-020-0448-0.

116. Ye Q, Wang B, Mao J, Fu J, Shang S, Shu Q, et al. Epidemiological analysis of COVID-19 and practical experience from China. J Med Virol. 2020; PG-. doi:10.1002/jmv.25813.

117. Yongchen Z, Shen H, Wang X, Shi X, Li Y, Yan J, et al. Different longitudinal patterns of nucleic acid and serology testing results based on disease severity of COVID-19 patients. Emerg Microbes Infect. 2020;9 1 PG-833–836:833–6. doi:10.1080/22221751.2020.1756699.

118. Yuan C, Zhu H, Yang Y, Cai X, Xiang F, Wu H, et al. Viral loads in throat and anal swabs in children infected with SARS-CoV-2. Emerg Microbes Infect. 2020;9 1 PG-1233–1237:1233–7. doi:10.1080/22221751.2020.1771219.

119. Yung CF, Kam KQ, Nadua KD, Chong CY, Tan NWH, Li J, et al. Novel coronavirus 2019 transmission risk in educational settings. Clin Infect Dis. 2020; PG-. doi:10.1093/cid/ciaa794.

120. Zachariah P, Johnson CL, Halabi KC, Ahn D, Sen AI, Fischer A, et al. Epidemiology, Clinical Features, and Disease Severity in Patients With Coronavirus Disease 2019 (COVID-19) in a Children’s Hospital in New York City, New York. JAMA Pediatr. 2020; PG-e202430:e202430. doi:10.1001/jamapediatrics.2020.2430.

121. Zachariah P, Halabi KC, Johnson CL, Whitter S, Sepulveda J, Green DA. Symptomatic Infants have Higher Nasopharyngeal SARS-CoV-2 Viral Loads but Less Severe Disease than Older Children. Clin Infect Dis. 2020; PG-. doi:10.1093/cid/ciaa608.

122. Zhang J, Litvinova M, Liang Y, Wang Y, Wang W, Zhao S, et al. Changes in contact patterns shape the dynamics of the COVID-19 outbreak in China. Science (80- ). 2020; PG-. doi:10.1126/science.abb8001.

123. Zhang L, Huang S. Clinical Features of 33 Cases in Children Infected With SARS-CoV-2 in Anhui Province, China-A Multi-Center Retrospective Cohort Study. Front Public Heal. 2020;8 PG-255:255. doi:10.3389/fpubh.2020.00255.

124. Zhang C, Gu J, Chen Q, Deng N, Li J, Huang L, et al. Clinical and epidemiological characteristics of pediatric SARS-CoV-2 infections in China: A multicenter case series. PLoS Med. 2020;17 6 PG-e1003130:e1003130. doi:10.1371/journal.pmed.1003130.

125. Zhang B, Liu S, Zhang J, Xiao J, Zhu S, Dong Y, et al. Children hospitalized for coronavirus disease 2019 (COVID-19): A multicenter retrospective descriptive study. J Infect. 2020; PG-. doi:10.1016/j.jinf.2020.04.045.

126. Zhao W, Wang Y, Tang Y, Zhao W, Fan Y, Liu G, et al. Characteristics of Children With Reactivation of SARS-CoV-2 Infection After Hospital Discharge. Clin Pediatr. 2020; PG-9922820928057:9922820928057. doi:10.1177/0009922820928057.

127. Zhao C, Xu Y, Zhang X, Zhong Y, Long L, Zhan W, et al. Public health initiatives from hospitalized patients with COVID-19, China. J Infect Public Heal. 2020; PG-. doi:10.1016/j.jiph.2020.06.013.

128. Zheng F, Liao C, Fan QH, Chen HB, Zhao XG, Xie ZG, et al. Clinical Characteristics of Children with Coronavirus Disease 2019 in Hubei, China. Curr Med Sci. 2020;40 2 PG-275–280:275–80. doi:10.1007/s11596-020-2172-6.
